# Conifers Phytochemicals: A Valuable Forest with Therapeutic Potential

**DOI:** 10.3390/molecules26103005

**Published:** 2021-05-18

**Authors:** Kanchan Bhardwaj, Ana Sanches Silva, Maria Atanassova, Rohit Sharma, Eugenie Nepovimova, Kamil Musilek, Ruchi Sharma, Mousa A. Alghuthaymi, Daljeet Singh Dhanjal, Marcello Nicoletti, Bechan Sharma, Navneet Kumar Upadhyay, Natália Cruz-Martins, Prerna Bhardwaj, Kamil Kuča

**Affiliations:** 1 School of Biological and Environmental Sciences, Shoolini University of Biotechnology and Management Sciences, Solan 173229, India; kanchankannu1992@gmail.com; 2 National Institute for Agricultural and Veterinary Research (INIAV), I.P., Vairão, 4485-655 Vila do Conde, Portugal; anateress@gmail.com; 3 Center for Study in Animal Science (CECA), ICETA, University of Porto, 4051-401 Porto, Portugal; 4 Scientific Consulting, Chemical Engineering, University of Chemical Technology and Metallurgy, 1734 Sofia, Bulgaria; msatanassova@abv.bg; 5 Department of Rasashastra and Bhaishajya Kalpana, Faculty of Ayurveda, Institute of Medical Sciences, Banaras Hindu University, Varanasi 221005, India; rohitsharma@bhu.ac.in; 6 Department of Chemistry, Faculty of Science, University of Hradec Kralove, 50003 Hradec Kralove, Czech Republic; eugenie.nepovimova@uhk.cz (E.N.); kamil.musilek@uhk.cz (K.M.); 7 School of Bioengineering & Food Technology, Shoolini University of Biotechnology and Management Sciences, Solan 173229, India; mails4sharmaruchi@gmail.com; 8 Biology Department, Science and Humanities College, Shaqra University, Alquwayiyah 11971, Saudi Arabia; malghuthaymi@su.edu.sa; 9 School of Bioengineering and Biosciences, Lovely Professional University, Phagwara 144411, India; daljeetdhanjal92@gmail.com; 10 Department of Environmental Biology, Sapienza University of Rome, Square Aldo Moro, 5, 00185 Rome, Italy; marcello.nicoletti@uniroma1.it; 11 Department of Biochemistry, University of Allahabad, Allahabad 211002, India; bechansharma@gmail.com; 12 School of Pharmaceutical Sciences, Shoolini University of Biotechnology and Management Sciences, Solan 173229, India; navneetqa@gmail.com; 13 Faculty of Medicine, University of Porto, 4200-319 Porto, Portugal; 14 Institute for Research and Innovation in Health (i3S), University of Porto, 4200-135 Porto, Portugal; 15 CESPU, Instituto de Investigação e Formação Avançada em Ciências e Tecnologias da Saúde, Rua Central de Gandra, 1317, 4585-116 Gandra PRD, Portugal

**Keywords:** conifers, phytoconstituent, oxidative stress, antibacterial, anti-inflammatory, anticancer, neurodegenerative

## Abstract

Conifers have long been recognized for their therapeutic potential in different disorders. Alkaloids, terpenes and polyphenols are the most abundant naturally occurring phytochemicals in these plants. Here, we provide an overview of the phytochemistry and related commercial products obtained from conifers. The pharmacological actions of different phytochemicals present in conifers against bacterial and fungal infections, cancer, diabetes and cardiovascular diseases are also reviewed. Data obtained from experimental and clinical studies performed to date clearly underline that such compounds exert promising antioxidant effects, being able to inhibit cell damage, cancer growth, inflammation and the onset of neurodegenerative diseases. Therefore, an attempt has been made with the intent to highlight the importance of conifer-derived extracts for pharmacological purposes, with the support of relevant in vitro and in vivo experimental data. In short, this review comprehends the information published to date related to conifers’ phytochemicals and illustrates their potential role as drugs.

## 1. Introduction

Medicinal plants have long been used as a source for traditional remedies in nearly all cultures [1]. Nature provides an endless supply of novel phytochemicals, which are referred to as natural products (NPs), and natural product drug development is a difficult task for developing new leads [2]. Traditional medicines (TMs) are valuable because they use natural products; for example, Ayurveda, Kampo, traditional Chinese medicine (TCM), traditional Korean medicine (TKM) and Unani use natural products and have been practiced for thousands of years, blossoming into well-regulated medical systems [3]. As time passed and medication progressed, synthetic drugs, such as enoxaparin, aspirin, warfarin, ibuprofen, naproxen, clopidogrel and diclofenac, became available over the counter and were linked to mild (headaches, back pain) to severe side effects (difficulty breathing, excessive bleeding, and hemorrhage) [4]. There are no doubts that the use of natural products has markedly improved certain forms of cancer, diabetes, hypertension, pain, memory deficit, Alzheimer’s disease (AD), and migraine [5], and their further use should be continued in order to meet the urgent need for effective drugs to treat human diseases [6].

**Figure 1:**
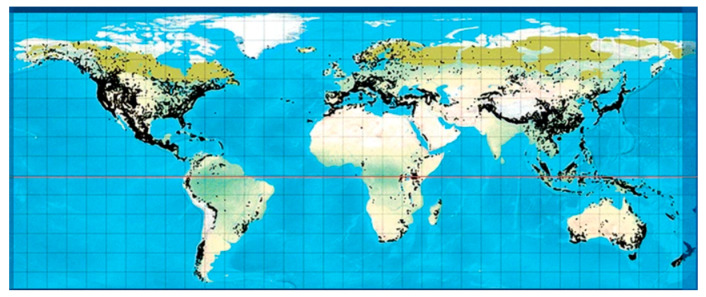
Global distribution of all conifers [11].

Coniferous plants, such as plants belonging to the *Taxus, Cupressus, Picea, Pinus, Cedrus, Araucaria* genera, are found worldwide and have shown several beneficial activities against diseases, highlighting the importance of conifers in drug development [7]. Conifers are woody, have needle-shaped single-veined leaves, and consist of male and female unisexual cones with bract scales [8]. They comprise eight families (*Pinaceae, Araucariaceae, Cupressaceae, Podocarpaceae, Cephalotaxaceae, Taxaceae, Phyllocladaceae, Sciadopityaceae*), 70 genera, and 630 species [9]. A number of genera include a vast number of species, such as *Pinus* (110), *Podocarpus* (105), *Juniperus* (55), *Abies* (50), *Picea* (35), *Dacrydium* (21), *Taxodium* (29) *Pseudotsuga* (22)*, Agathis* (22), *Araucaria* (19), and *Taxus* (19), whereas there are some genera which contain a lower number of species, including *Larix* (10), *Cedrus* (4), *Torreya* (6) and *Cryptomeria* (1) [10]. They can be found in abundance in tropical lowland and submontane forests (Figure 1).

**Figure 2:**
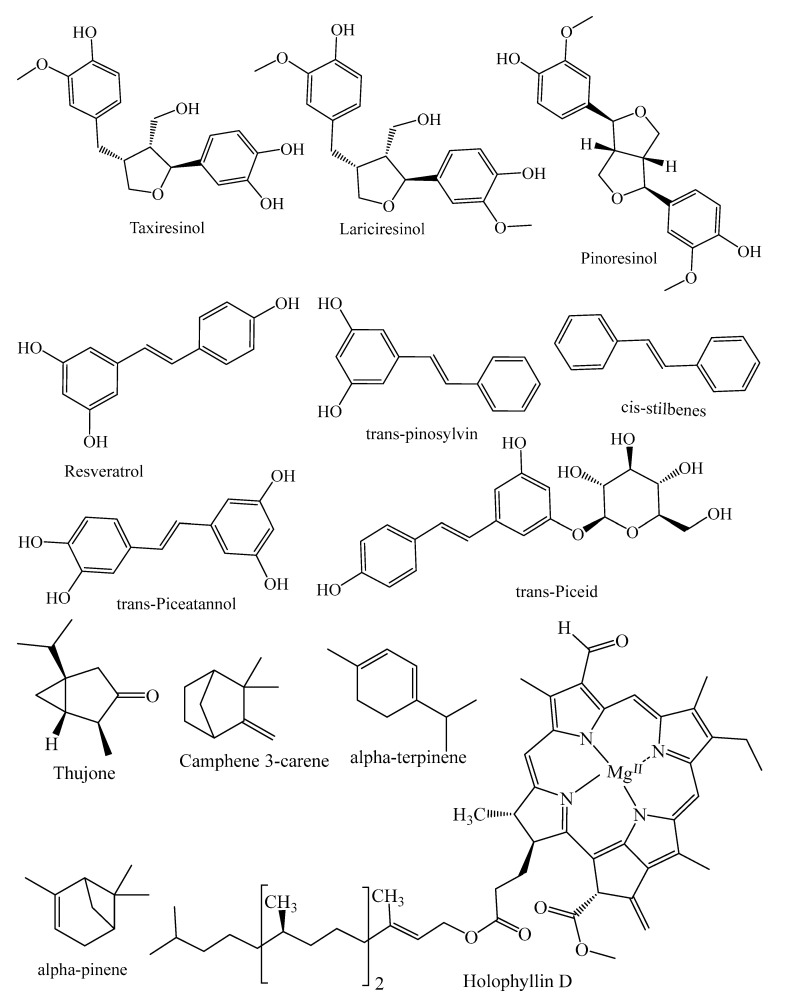

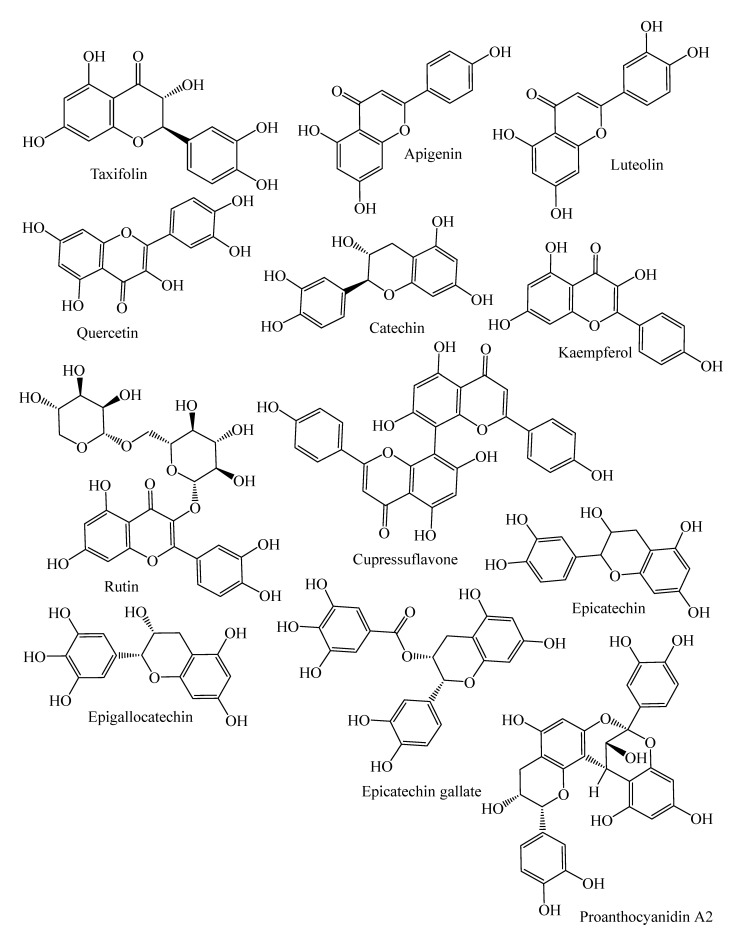

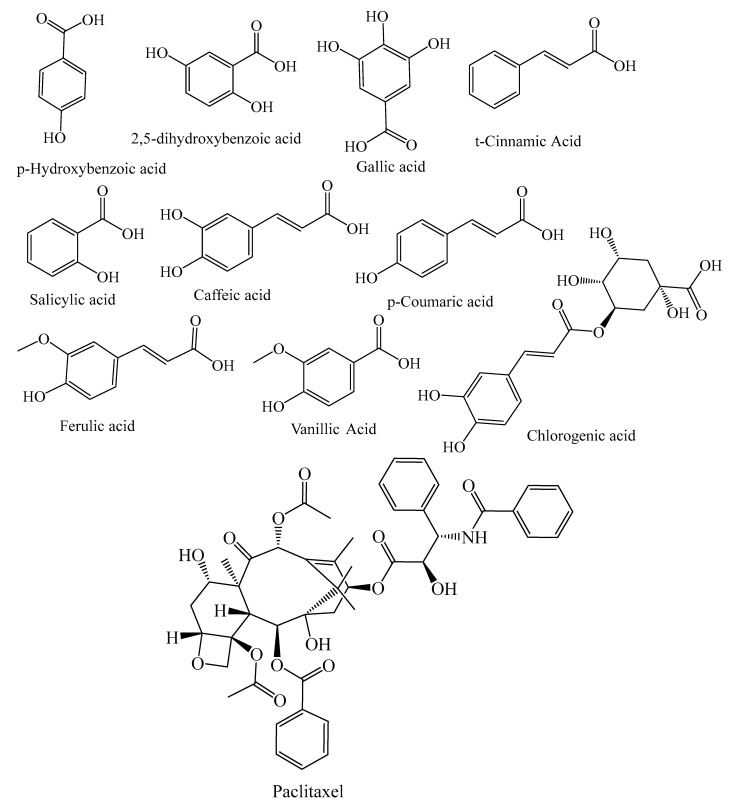
Structures of phytochemical compounds present in different conifer spp.

They contain secondary metabolites that combat pathogens and activate the plant’s defense mechanism [12]. The three major phytochemical groups, viz. terpenoids (resin acids and terpenes), alkaloids (piperidines) and polyphenols (phenolic acids, flavonoids, proanthocyanidins, lignans, acetophenones, and stilbenes) [13], present in the species of conifer trees are shown in Table 1, and their phytochemical compounds’ structures are shown in Figure 2. It is very important to understand the evolutionary pathway of Gymnosperms, in accordance with Sporne’s proposal, wherein the conifers represent the core, and the teachings that we can receive from it, comparing the morphological characters and their correlation with the chemical ones [14].

**Table 1:** Phytochemical constituents present in conifers.

Subclasss	Main Examples of Each Class	Conifer spp.	References
**Terpenes**			
Monoterpenes	β-myrcene, α-pinene, limonene, α-terpinene, thujone, camphene, β-pinene, thujole, Δ-3-carene, phellandrene	*Pinus roxburghii, P. pinea, P. wallichiana, P. pinaster, P.sylvestris, P. gerardiana, P. nigra, P. radiata, Thuja occidentalis, Abies alba, Picea abies, Metasequoia glyptostroboides*	[12,15,16,17]
Sesquiterpenes	Laurenobiolide, farnesene, inulicin, vernodalin, 3*H*-benzofuaran-2-one, 4-methyl-3-methoxy-3*H*-benzofuaran-2-one, 4,9(α)-dihydroxynardosin-6-en, delta-cadinene, alpha-humulene, beta-cedrene, trans-caryophyllene, cubenol	*P. mariana, Juniperus foetidissima, A. alba, P. abies, M. glyptostroboides, J. phoenicea, P. roxburghii*	[8,12,18,19,20]
Diterpenes	Paclitaxel, 10-deacetylbaccatin III, tasumatrol B, taxodal, sandaracopimaric acid, taxodione, xanthoperol, andrographolide, gibberellin A8, 7α-hydroxysandaracopimaric acid, gibberellin A12, gibberellin A12 aldehyde, gibberellin A15, 14α-hydroxyisopimaric acid, 12-hydroxydehydroabietic acid, gibberellin A19, gibberellin A9, carnosol, lathyrol, *E*-communic acid, 15-hydroxy-8(17),13(*E*)-labdadiene-19-carboxilic acid, holophyllin A, holophyllin D, sugiol, ferruginol	*Taxus brevifolia, T. baccata, T. globosa, T. distichum, P. mariana, J. taxifolia, M. glyptostroboides, A. holophylla, J. excelsa, J. communis, J. excelsa, J. communis*	[18,19,21,22,23,24,25,26,27]
**Nitrogen Compounds**			
Alkaloids	Vellosimine, 1,6-dehydropinidine, cis-pinidine, 1,6-dehydropinidinone, epipinidinone, cis-pinidinol, trans-pinidine, euphococcinine, α-pipecoline 1, (−)-pinidine	*P. mariana, P. abies, P. sabiniana, P. torreyana,*	[13,18,28]
Lignans	Lariciıesinol, taxiresinol, 3’-demethylisolariciresino1-9’-hydroxyisopropylethe, isolariciresinol, deoxypodophyllotoxin, (−)-secoisolariciresinol, 3, 3-demethylisolariciresinol, isotaxiresinol 2, α-conidendrin, (+)-pinoresinol, (−)-matairesinol, arctiin, dibenzylbutyrolactol, (−)-wikstromol, (−)-traxillagenin, (−)-arctigenin, traxillaside, 4′-deme-thyltraxillagenin, [(2*R*,3*R*)-2-(4′’-hydroxy-3′’-methoxybenzyl)-3-(4′-hydroxy-3′,5′dimethoxybenzyl)-butyrolactone]	*T. baccata, J. taxifolia, J. sabina, J. virginiana, J. virginiana, P. roxburghii, Cedrus deodara, T. nucifera*	[29,30,31,32,33,34,35]
**Polyphenols: Flavonoids**			
Flavanonols	Taxifolin, cedeodarin	*C. deodara, L. simbraca, P. roxburghii, P. mariana, P. abies, A. pindrow, A. excelsa; P. pinea, P. halepensis, P. pinaster, P. gerardiana*	[33,36,37,38,39,40]
Flavones	Pilosanol B, luteolin, apigenin, apigenin 6-C-b-glucopyranoside	*P. mariana, A. excelsa, P. abies, P. sylvestris, P. menziesii, P. menziesii, J. communis, A. angustifolia, L. deciduas*	[18,38,39,41,42,43]
Biflavones	Bilobetin, cupressuflavone II-7-*O*-methyl-robustaflavone	*T. wallichiana, C. macrocarpa, A. angustifolia*	[43,44,45,46]
Flavonols	Quercetin, dihydroquercetin, rutin, kaempferol, dihydrokaempferol	*J. communis, J. oxycedrus, P. gerardiana, P. roxburghii, P. wallichiana, A. angustifolia, P. abies, L. deciduas, P. sylvestris, P. menziesii, M. glyptostroboides, J. excelsa, P. mariana, J. foetidissima*	[18,33,41,42,43,47,48,49,50]
Flavan-3-ols	Monomers: (−)-epicatechin, (−)-epicatechin-3-gallate, (+)-catechin, sennidin A, (−)-epigallocatechin,	*P. pinaster, P. pinea, P. halepensis, P. roxburghii, P. wallichiana, P. gerardiana, J. foetidissima, A. angustifolia, P. abies, L. deciduas, P. sylvestris, J. communis, P. menziesii, J. oxycedrus, M. glyptostroboides, J. excelsa*	[18,33,39,40,41,43,47,48,49,50]
	Polymers: Procyanidin B1, B2, procyanidin A2,	*P. halepensis, P. pinea, P. pinaster*	[40]
**Phenolic acids**			
Benzoic acids	p-hydroxybenzoic acid, 2,5-dihydroxobenzoic acid, gallic acid, 4-hydroxybenzoic acid, protocatechuic acid, ellagic acid	*P. abies, L. deciduas, P. sylvestris, P. menziesii, P. kesiya, J. communis, A. excelsa, P. roxburghii, P. wallichiana, P. gerardiana, L. deciduas, J. communis*	[33,38,41]
Hydroxycinnamic acid	Caffeic acid, t-cinnamic Acid, p-coumaric acid, vanillic acid, ferulic acid, salicylic acid, sinapic acid, syringic acid, chlorogenic acid, 5-caffeoylquinic acid, caffeic acid 4-*O*-glucoside	*P. abies, L. deciduas, P. sylvestris, T. baccata, P. mariana, P. pinaster, P. kesiya, L. deciduas, J. communis, P. menziesii, M. glyptostroboides*	[18,21,39,41,42,49]
Stilbenes	trans-resveratrol, resveratrol, trans-pinosylvin, cis-stilbenes, pinosylvin, dihydro-monomethyl, trans-stilbenes, trans-piceatannol, trans-piceid, trans-isorhapontin, trans-isorhapontigenin, phenanthrenes, astringin, trans-astringin	*P. mariana, P. abies, J. communis, P. pinaster, P. sylvestris, P. strobes, P. roxburghii, P. wallichiana, P. gerardiana, P. merkusii*	[8,18,39,51,52,53,54,55,56,57]

It is also important to keep in mind the strong tendency towards adaptation, certainly not comparable with that of the subsequent Angiosperms, of which the dominance of the Araucaceae in the southern part of South America is a shining example [58]. Once again, it appears evident that the climatic situation constitutes the determining factor in the evolutionary path, as evidenced by the residual dominance of the conifers in the environments suited to them. There is therefore a lot to explore relating to conifers, and it is useful to develop studies entirely dedicated to individual genera [59].

From the chemotaxonomic point of view, it is necessary to highlight how difficult it is to study the chemistry of conifers due to the lack of easily usable markers [60]. In fact, resin and phenolic compounds, including tannins, have proved, due to their complicated composition and wide variability, difficult to study and not suitable for deducing clear considerations from them. First of all, we must not forget the unsuccessful attempts, the once well-developed branches that are now essentially exhausted, of which *Ginkgo* is a sensational example, to which we can add the genus *Taxus* [61]. It is no coincidence that these species are now a source of drugs of great therapeutic importance. It is therefore possible that there is still a lot to investigate and study on the chemistry of conifers. A starting point is represented by the collection of the current scientific knowledge recorded so far and the possible use of these data in the light of the most recent interpretations and possible therapeutically interesting utilizations. In this sense, the main focus of this review is to emphasize conifers’ phytochemical compounds with a broad range of applications and as a source of molecules for drug development.

## 2. Conifers Phytochemicals Components

### 2.1. Terpenes

Terpenes are isoprenoids not containing nitrogen and sulfur and seem to be the main and largest group of natural phytochemicals group in conifers [12]. The terpenoids can be classified as C5 (hemiterpenes), C15 (sesquiterpenes), C20 (diterpenes), C10 (monoterpenes), C25 (sesterpenes), C40 (tetraterpenes), C40 (polyterpenes), and C30 (triterpenes) on the basis of C5 units [62]. Table 1 represents the most common mono-, sesqui- and diterpenes present in conifers. In particular, monoterpenes have been extensively studied, especially for their antiviral properties. Further, Porres-Martínez et al. (2016) reported their biological activities, including the anti-inflammatory, anticancer, antioxidant, and neuroprotective effects [63]. However, taxol diterpene derived from *Taxus* spp. have potential against malaria and cancer [62]. Sesquiterpenes have antiseptic, antimicrobial and disinfectant properties [64]. Kopaczyk et al. (2020) showed that the antioxidant activity of terpenes can prevail over oxidative stress aggravated by internal and external stimuli [12].

### 2.2. Alkaloids

There are several classes of alkaloids which are classified on the basis of the heterocyclic ring system and biosynthetic precursor which are of great interest. The alkaloids comprise quinolizidines, indoles, tropanes, pyrrolidines, pyrrolizidines, imidazoles, piperidines and isoquinoline purines [65]. There are numerous studies on the biological activity and medicinal uses of alkaloids [66]. In addition, alkaloids have been shown to have antitumor, anti-hyperglycemic and antibacterial activities [13]. Virjamo et al. (2020) reported that among the piperidine compounds of *P. abies*, only 1,6-dehydropinidine exhibited antibacterial effects by using a larger number of strains, whereas *cis*-pinidine was revealed to be toxic for vertebrates, which may only act in defense against herbivores [13].

### 2.3. Polyphenols

Polyphenols are of major relevance and perform a range of functions from skeletal constituents in various tissues to pigmentation in many plant organs [67]. They act as natural antioxidants, being able to inhibit lipid peroxidation, carcinogenesis, antimicrobial activity, direct capillary constrictive action, phytohormones, and have also the ability to stabilize ascorbic acid [68]. Flavonoids (isoflavones, flavonols, flavanonols, flavones, tannins, flavanones, anthocyanidins), stilbenes (resveratrol), phenolic acids (hydroxybenzoic and hydroxycinnamic acids), lignans, can all be found in plants [69]. Polyphenols, especially flavonoids, such as rutin, quercetin, apigenin, and epicatechin, are widely found in conifers. The genera *Araucaria, Pinus, Cedrus*, etc. are reported for their antimicrobial, anticancer, antidiabetic, neuroprotective [43] and anti-inflammatory properties and can be used in the treatment of neurodegenerative diseases, as well as being helpful in reducing αβ toxicity and neuronal dysfunction [70].

#### 2.3.1. Flavonoids

Flavonoids are the most abundant phenol group in nature, present in a wide range of conifers [71]. Flavonoids have a central three-ring structure, but the different subclasses vary due to the centrally located heterocyclic ring structure (C-ring), which connects the two benzene rings [72]. To date, more than 6000 flavonoids have been recorded in several studies from plants. Flavonoids are aglycones in their basic structure, but most of them are glycosides in plants [73]. The subclasses of flavonoids found in the leaves, barks and seeds of conifers are represented in Table 1.

#### 2.3.2. Lignans

Lignans are phenylpropanoids dimers made up of two coniferyl or sinapyl alcohol units bound together at the tails [74]. Isolariciresinol, taxiresinol, lariciresinol, pinoresinol, and their glycosides are examples of such compounds. There is a growing interest in lignans, especially because of their chemotherapeutic ability [75]. The most commonly present lignin compounds in conifer spp. are shown in Table 1.

#### 2.3.3. Stilbenes

Stilbenes are produced by a number of conifer species, including *Pinus sylvestris* and *Picea abies*. Briefly, stilbenes are phenolic compounds with a heterologous bridge connecting two aromatic rings [76]. Many other compounds, such as trans-pinosylvin, cis-stilbene, resveratrol and piceatannol, have been isolated from the barks of conifer species (Table 1). For stilbenes, excellent antimicrobial effects have been reported [71].

#### 2.3.4. Tannins

Tannins are polyphenolic compounds that can be in a wide range of plants. Tannins are colored pigments, astringent and are characterized by a bitter taste [77]. Tannin-rich conifer bark extracts have antimicrobial properties and high potential in preventing lipids from oxidation in the liposome model [78]. As a result, the tannins can be divided into four main classes based on their structural characteristics: gallotannins, complex tannins, ellagitannins and condensed tannins [79]. Condensed tannins (CTs), also well-known as proanthocyanidins, are prevalent in *P. abies* and *P. sylvestris* tree bark [77]. Condensed tannins are considered as polymers or oligomers of flavan3-ol units connected by C-C bonds that are hydrolysis resistant [80]. Procyanidins (PCs) and prodelphinidins (PDs) are the most popular PAs. Catechin and other epicatechin units make up PCs. Epigallocatechin units make up PDs [81].

Tannins exhibit antioxidant activity through various pathways, including free radical scavenging, transition metal chelation, and inhibition of pro-oxidative enzymes [82], besides having the capability to bind and form complexes with proteins and other compounds, and being responsible for their biological activity [83]. Tannins also act as antimicrobial agents, inhibiting extracellular microbial enzymes, depriving microbial growth substrates, and exerting a direct action on microbial metabolism, such as the denaturation of cell membrane proteins [84]. In the food industry, they could be used as functional coatings, adhesives, preservatives and as flavor compounds [71]. In a study, pine and spruce bark-derived PA-rich extracts revealed good potential for use in the food industry to develop preservative agents and to prevent lipid peroxidation in food items containing fatty-acids [78].

## 3. Traditional Medicinal Uses

Since prehistoric times, coniferous plants have been used as a medicinal source. Plant-based research has received more attention in recent years, and the literature supports the possible use of medicinal plants in conventional processes [85]. *T. orientalis* leaves and stems are utilized in traditional medicine to cure nervous system disorders, insomnia, heart palpitations, hemorrhage and fever. Fresh cedar leaves steeped for seven days in a 60% alcohol solution are often used by traditional Chinese physicians to encourage hair growth [86]. *Cupressus* spp. leaves, cones, stem bark have also been revealed to be useful in the treatment of hemorrhoids, bleeding varicose veins, asthma cough, spasms, diarrhea, rheumatism, common colds, piles, urinary tract ailments and vaginal discharge [7]. Different parts of the *Pinus* spp. bark, leaf, cone, and resin are also prescribed to treat cold-influenza, cough, tuberculosis, and bronchitis as a diaphoretic, rubefacient, antiseptic, diuretic, stimulant and febrifuge, while resin is also used in wound healing and injury [87]. The extract from *Juniperus* spp. leaves, berries and bark has also been used for the treatment of chronic eczema, hyperglycemia, obesity, tuberculosis, bronchitis, and pneumonia. The female cones, wood and leaves of *J. foetidissima, J. communis* and *J. excelsa* are used as a tonic for gout and rheumatism, a carminative, a diuretic, a treatment for urinary tract infection and stomach ache, an expectorant, a stimulant, an emmenagogue, and a treatment for the common cold [7].

Different parts of *Taxus* spp. have precise ethnomedicinal uses; for example, the leaves’ juice is used to cure cancer and bronchitis; bark juice and other parts, such as the leaf, are used for asthma and bronchitis, while trunk oil and cones are used to treat sheep diseases, bad breath, halitosis, inflammatory diseases of the lower urinary tract, renal stones, urinary infection, rheumatism dyspeptic complaints, hemorrhoids and cancer [7,88], and powdered dried leaves are considered to be effective in epilepsy, asthma, headache, diarrhea, bronchitis and hiccoughs [89]. A decoction developed from the bark is used to relieve pain from the muscles, knees, and rheumatoid arthritis, whereas a decoction made from the leaves is used to treat liver issues [90].

## 4. Conifers Extracts Rich in Phytochemical with Putative Health Effects

### 4.1. Oxidative Stress

**Figure 3:**
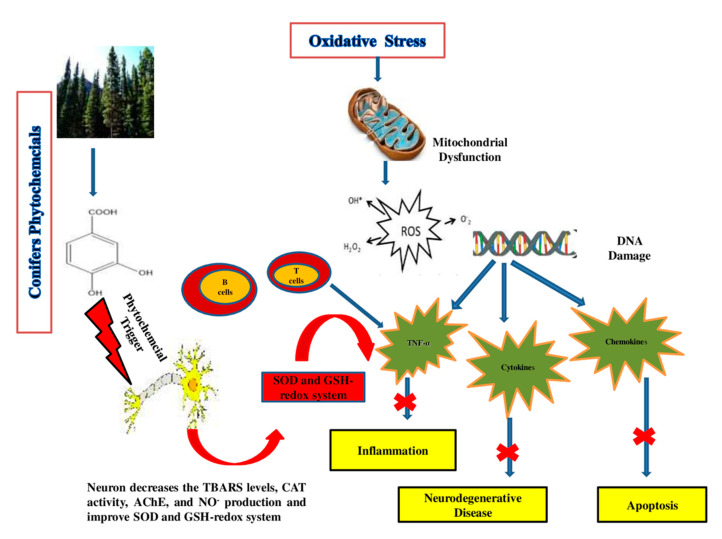
Action mechanism of conifers’ phytochemical compounds in oxidative stress, apoptosis, and neurodegenerative diseases. The phytochemicals’ multi-target effects in the brain include mitochondrial protection, anti-aggregation, anti-oxidant, anti-apoptotic and anti-inflammatory activity.

The role of oxidative stress in the progression of degenerative ageing is well understood. Diabetes mellitus, coronary heart disease, cancer, inflammation, stroke, neurological conditions (e.g., AD), and ageing have all been linked to reactive oxygen species (ROS) generation [49]. Both enzymatic and non-enzymatic reactions are involved in the synthesis of ROS. ROS are known to be generated by enzymatic reactions present in many cell processes, including the respiratory chain system, prostaglandin synthesis and phagocytosis [91]. All ROS are produced through enzymatic action, including NADPH oxidase, xanthine oxidase and peroxidase in many cell processes, in whom the superoxide radical (O_2_^●−^) is generated [92]. Different ROS, formed during enzymatic reactions and with the action of enzymes (amino acid oxidase and xanthine oxidase), include hypochlorous acid (HOCl), hydrogen peroxide (H_2_O_2_), peroxynitrite (ONOO-), and hydroxyl radicals (OH^●^) [93]. The “Fenton reaction” between O^2−^ and H_2_O_2_ takes place in the presence of Fe^2+^ or Cu^+^, which work as catalysts, producing OH, the most reactive free radical species [94]. The production of ROS has been related to non-enzymatic interactions between oxygen and organic compounds, as well as when cells are in contact with ionizing radiation during mitochondrial respiration [94,95]. Extensive research is currently needed to discover phytochemical compounds with the ability to boost the immune system and reduce oxidative stress [96]. The quest for new antioxidant molecules is a crucial part of promoting healthy ageing and combating oxidative stress [93]. Flavonoids, phenolic acids, vitamins and carotenoids are examples of natural compounds with antioxidant potential that have antagonistic effects on degenerative and inflammatory processes throughout the body, have beneficial effects on the immune and digestive systems, prevent ROS-related chronic problems and improve the overall quality of life, as shown in Figure 3 [88].

### 4.2. Synergism between ROS and other Diseases

The overproduction of ROS has been linked to a variety of chronic diseases, including cardiovascular, inflammatory and neurodegenerative diseases, and even cancer. The following sections explain on the relationship between ROS and chronic diseases.

#### 4.2.1. Antioxidant Activity

In chronic obstructive pulmonary diseases (COPD), ROS play an important role [97]. The bark, wood, needle, and cone extracts of the *Pinaceae* family are high in polyphenolic compounds (Pycnogenol), primarily procyanidins, stilbenes, tannins and phenolic acids, and have significant antioxidant activity against ROS [98]. The antioxidant activity of conifer extracts has been confirmed by DPPH, FRAP and reducing power assays [51].

Flavonoids’ ability to scavenge free radicals can protect the human body from oxidative damage, which accelerates the ageing process [99]. Pycnogenol^®^, a polyphenol-rich compound extracted from the bark of *P. pinaster, P. glauca,* and *P. mariana*, has shown the ability to boost plasma antioxidant capacity and ameliorate pulmonary function and asthma traits [51,100]. In a study by Senthilmohan et al. (2003), 6–12 weeks of supplementation of Enzogenol^®^, and proanthocyanidin-rich flavonoid extracted from *Pinus radiata* bark in combination with vitamin C reduced DNA and protein oxidative damage in 55–75-year-old people [101]. In vivo studies have reported that the intake of quercetin alone did not protect DNA, but the combination of flavonoids (quercetin and myricetin) and isoflavonoids provides protection against DNA damage [102]. It has been found that the stilbenoid component resveratrol and piceatannol obtained from conifer spp. have more potent biological activities, namely as antioxidants [103]. Terpenoids and phenolic compounds, such as pinene, lycopene, camphene, gallocatechin, lutein, limonene and catechin found in the extract of conifer bark and needles have also been reported for their antioxidant potential by DPPH, FRAP, H_2_O_2_, ABTS assays, as shown in Table 2 [104].

#### 4.2.2. Anti-Inflammatory Activity

When contagious microorganisms such as fungi, bacteria and viruses come into contact with the body, they remain in specific tissues and flow into the bloodstream, causing inflammation [105]. This also occurs as an end result of tissue damage, cancer, cell death, degeneration and ischemia [106,107,108]. In most cases, both the innate and adaptive immune responses are responsible for inflammation development [109]. The primary protection against invading foreign microbodies and cancer cells is the innate immune system, which involves macrophages, dendritic cells, and mast cells [105]. In the adaptive immune system, specialized cells (B and T cells) remove foreign pathogens and cancer cells by generating specific receptors and antibodies [110]. Cytokines such as interleukins, interferons, tumor necrosis factor, eicosanoids (leukotrienes and prostaglandins), modulatory inflammation-transcription nuclear factor (NF-ĸB) and chemokines (monocyte chemoattractant protein 1), are the inflammatory mediators and cellular pathways that have been extensively studied in relation to human pathological conditions [111]. Tumor necrosis factor-α (TNF) is a pro-inflammatory cytokine that is secreted by a variety of cells and has a variety of cellular effects [112]. It has also been linked to a variety of human illnesses, including cancer, mental and skin disorders, immune and inflammatory diseases. IL-1 is another cytokine that primarily has a pro-inflammatory effect [113]. It raises the levels of pro-inflammatory cytokines, including IL-1, TNF and IL-6 [114]. On the other hand, IL-1 has been linked to anti-inflammatory properties. Likewise, IL-1α and IL-6 originating from activated mast cells in the innate immune response also boost acute phase protein synthesis and thus show some anti-inflammatory effects [115]. The cytokine family members, including IL-12, IL-27, IL-23 and IL-35, function as a pro- and anti-inflammatory response [111,116,117]. On the other hand, IL-10 has been recognized as an effective anti-inflammatory cytokine, and helps in preventing several pro-inflammatory mediators from further action [118]. It protects tissue from homeostasis, defends against injury and damage caused by an overactive inflammatory response [118,119,120]. TNF-α accelerates PGE2 synthesis changes caused by edema and the flow of blood [46]. The extraction of plant materials is the first step in deciding the plant biological activities. The is a high probability of synergism between bioactive components when a whole extract is used, which could be lost if each and every component is isolated [121]. This form of synergism has been documented in numerous medical studies, generally for anti-inflammatory function [105]. There are different types of extraction and separation processes, such as:

1. Soxhlet extraction: The Soxhlet extraction method is a more efficient extraction method with high extraction yield and requires less solvent and time. This method requires electricity and solvents such as methanol, petroleum ether, and acetonitrile for the extraction process. However, sometimes high temperature and long extraction time enhance the possibility of thermal degradation and the loss of bioactive compound fraction activity [122]. 

2. Percolation: Extraction yield is better in percolation than maceration; in this process, pre-soaked plant material is added to a container, which allows the constantly controlled removal of the extract via a valve at the bottom and adding fresh solvent from the top.

3. Maceration: Maceration is carried out at room temperature by soaking the material with the solvent with eventual stirring. It has the advantage of moderate extraction conditions but suffers from high solvent consumption, long extraction times and low extraction yields. It could be used for the extraction of thermo labile components. 

4. Ultrasound-assisted extraction: In UAE, the plant material, usually in a glass container, is covered by the extraction solvent and put into an ultrasonic bath. It decreases extraction time and improves extraction yields due to mechanical stress, which induces cavitation and cellular breakdown, and has gained increasing popularity [123]. For the isolation of extract from the solvent, the distillation process and many evaporators are used. After isolation to concentrate the extract, many researchers used a rotary evaporator, a normal air-drying process and distillation methods. Generally, to separate different solvent extracts, a separatory funnel is required [124].

Anti-inflammation is one of the main recorded effects of conifer phytochemicals among the numerous biological activities that have been studied so far. Table 3 reported the anti-inflammatory effect of conifer phytochemicals in in vivo and in vitro models.

Cupressuflavone (CUF) isolated from *C. macrocarpa* has the ability to reduce the levels of several cytokines, including IL-1b, IL-6, TNF-α and PGE2, in plasma dose-dependently, and thus acts as an anti-inflammatory agent [46]. Triterpenoids and abietane type’s compounds extracted from *Abies chensiensis* show anti-inflammatory effects against NO production in RAW 264.7 macrophage cells [125]. It has been found that tasumatrol, deacetylbaccatin, paclitaxel and many other terpenoids extracted from *Taxus* spp. are effective in the anti-inflammatory process initiated through the carrageenan and cotton pellets induced edema model [21,22]. Kyung-Jae Cha. (2016) reported that in atopic dermatitis, the *Picea wilsonii* mast extract is useful and potent only in the inhibition of the production of the inflammatory cytokines IL-6, MCP-1 and IL-13, without significant change in IL-8 production induced in human adult low-calcium high-temperature (HaCaT) cell lines [115]. *T. occidentalis* mother tincture-containing terpenoids (thujone), polyphenols and flavanoids have potential in reducing ulcerative colitis inflammation in the mouse intestine and rectum by decreasing the stimulation of the pro-inflammatory cytokines IL6 and TNF-α induced by 2,4,6-trinitrobenzenesulfonic acid (TNBS) [126]. THP-1 cell adhesion to TNF was suppressed by enzogenol at a concentration of 5–25 g/mL onto TNF-α-activated human umbilical vein endothelial cells (HUVEC) by reducing integrin β2 induction and inhibiting monocyte trans-endothelial migration [127]. The anti-inflammatory and platelet-inhibitory effects of pycnozenol, extracted from *Pinus maritime* bark extract, inhibited the activity of cyclooxygenase (COX)-1 and COX-2 present in human plasma [128]. Inflammation has been attributed to cancer and neurodegenerative diseases [46].

#### 4.2.3. Anticancer Activity

According to the report by the World Health Organization, cancer was a major cause of death in 2018, with a death rate of 9.6 million people [129]. Hippocrates, before 370 B.C., coined the word “cancer” to describe carcinoma tumors [130]. On the basis of evidence, bone cancer was identified in Ancient Egyptian mummies in around 1600 B.C., and cancer of the breast was identified in 1500 B.C., although there is no record of a cure for cancer [131]. Giovanni’s research laid the foundation for scientific cancer techniques in 1761, when he performed the first autopsies on dead human bodies to determine the connection between a patient’s disease and their death, as well as pathologic studies [132]. Cancer has been identified as the chief matter of public health concern around the world [133]. Surgery, radiotherapy, and chemotherapy are some of the conventional cancer treatments [134,135]. On the other hand, despite the use of a variety of synthetic drugs for cancer treatment and the successful completion of different management schedules, current therapies are not able to achieve the desired results, as tumor relapse and metastasis are common [136]. Nature contains various chemicals and pharmacologically active substances which act as anticancer drugs [137]. Recently, many of the phytochemicals and synthetic analogs, such as HS-1793 (resveratrol), have been identified as inhibiting the growth of cells and inducing apoptotic cell death, helping to cure cancer [138]. While only a few phytochemical compounds obtained from natural products have been developed into clinically active drugs, their bioactive components may be used as a basis for the development of more successful analogues and prodrugs using chemical techniques such as metabolomics, complete or combinatorial fabrication, and biosynthetic pathway modification [139]. Many phytochemical compounds are highly efficient in inducing apoptosis and cytotoxicity by modulating different MAPK andAKT/PI3K pathways, and suppress cancer cells line invasion and migration potential along with the stimulation of senescence phenotype, regulation of Bax or p53 genes, cell cycle arrest and modulation of IL-8, IFN-γ, TNF, IL-6 [140]. Many of the compounds derived from conifers’ bark and leaves act as antitumor drugs, such as paclitaxel (PTX) (trade name Pycnogenol^®^ and Taxol^®^), a diterpene found in the crude extract of *P. pinaster* and *Taxus brevifolia* bark [141]. Paclitaxel, as well as its analogues docetaxel (taxoteres) and jevtanas (cabazitaxel), are examples of chemotherapeutic synthetic analogues derivative from plants that have been formulated and validated clinically [139]. By binding microtubules, PTX and other microtubule-targeting agents (MTAs) induce cellular death [142]. Microtubules are tubulin heterodimers that play a role in disease and perform numerous cellular functions including transport, force production in cell division, and structural support [143]. During the G2 phase of the cell cycle, tubulin is produced, and microtubules are assembled. Microtubule stabilizing agents, such as PTX, bind to α/β tubulin in order to disassemble microtubules. As a result, they cause cell death and are used as an anti-cancer agent [144]. In general, cells exposed to PTX are stuck in the G2/M phase, resulting in death due to failure to move through the cell cycle [145,146]. Recently, a study published in 2017 found that pycnogenol and PTX at doses of 20 g/mL and 0.5 μM cause DNA and mitochondrial damage in cancer breast cell line (MDA-MB) in 24 h, and concluded that it is possibly a target drug for cancer treatment through DNA and mitochondrial damage mechanisms [141]. From different conifer species crude extracts, the anticancer activities on different cancer cell lines are summarized in Table 4.

**Table 2:** Antioxidant capacity of extracts obtained from different conifer spp.

Conifer spp.	Part Used	Compounds	Nature of Extract	Radical Scavenging Assay	Dose/Concentration	Main Effects	References
*Aurocaria cookii*	Leaves	Phenolic compounds	Methanol, chloroform, petroleum ether	DPPH	1000 μg/mL	Methanol extract shows the best antioxidant activity with 63% inhibition, higher than the other two compounds	[147]
*A. excelsa*	Needle	Flavanoids	Methanol	DPPH	50–72.5 μg/mL	Methanol/water extract shows antioxidant activity	[38]
*C. deodara*	Heart wood	Tannins, flavonoids, and phenolic compounds	Water/alcohol	DPPH, superoxide radical-scavenging activity, ABTS	DPPH-IC_50_ (μg/mL): 61.89 (water extract), 75.79 (alcohol extract) superoxide radical-scavenging activity— IC_50_ (μg/mL): 87.76 (water extract), 121.55 (alcohol extract). ABTS-IC_50_ (μg/mL): 115.29 (water extract), 122.42 (alcohol extract).	DPPH radical-scavenging activity and the reducing power of *C. deodara* were potent in water and alcohol extract	[148]
*C. japonica*	All parts	Phenolic compounds	Methanol	ORAC, SOD	4.09–7.64 TE/mg 3.63–4.06μg/mL	The methanol extracts from each part of *C. japonica* except for pollen showed strong activities in the bioactivity assays.	[149]
*J. communis*	Berry	Flavanoids (quercetin rutin, apigenin) chlorogenic acid	Alcohol/Water	DPPH	EC_50_ 1.42 mg/mL against standard Ascorbic acidEC_50_ value of 0.365 mg/mL	The antioxidant activity was confirmed as 81.63 ± 0.38% by the DPPH assay.	[42]
*L. laricina*	Bark	Phenolic compounds	Ethanol/Water	ORAC	IC_50_ 0.878 μg/mL.	Bark extract of LL shows significant antioxidant activity	[51]
Metasequoia glyptostroboides	Cone	Terpenoid	Ethyl acetate	DPPH, NO, superoxide, and H_2_O_2_	5–250 μg /mL	Sugiol derived from cone extract show good antioxidant activity—78.38, 72.42, 74.45 and 85.04%, respectively.	[26]
*Picea abies*	Bark	Atilbenoids	Ethanol/Water	DPPH	49.74 μg/mL	UVA-induced modification of the stilbene-rich inner bark extracts increased the antioxidant activity as UVA irradiation decreased the capacity of the extracts to prevent lipid oxidation in the liposome system method	[53]
*P. smithiana*	Leaves	Phenolic compounds	Methanol	DPPH	IC_50_ (μg/mL)-	Results of the DPPH radical scavenging activity and FRAP study determine that methanol extracts of leaf displayed the highest antiradical efficiency	[150]
					228		
				FRAP	494		
				Reducing Power assay	978		
*Pinus gerardiana*	Bark	Phenolic compounds	Ethanol	DPPH	IC_50_ value μg/mL	*P. gerardiana* shows promising H_2_O_2_ radical scavenging activity	[104]
					102.8		
				H_2_O_2_	81.83		
				NO_2_	109.2		
*P. halepensis*	Bark	Phenolic compounds	Ethanol/Water		IC_50_ (μg/mL). Ethanol and the water	Ethanol and water extract of bark exhibited significant free radical neutralization capacities, at conc. 0.5–8 μg/mL	[151]
				DPPH	3.28, 3.26		
				ABTS	3.1, 3.59		
*P. pinaster*	Bark	Phenolic compounds	Ethanol/Water		PB (50%) and (90%) IC_50_ value μg/mL	PP bark extracts formed from PB 50% (50% ethanol) have maximum (DPPH, ABTS) radical scavenging activity while FRAP shows activity with (PB 90%)	[39]
				DPPH	49.74		
				ABTS	59.41		
				FRAP	101.3		
*P. roxburghii*	Bark	Phenolic compounds	Ethanol		IC_50_ value μg/mL	Pine extract shows significant antioxidant activity	[104]
				DPPH	97.54		
				H_2_O_2_	86.90		
				NO2	111.38		
*P. wallichiana*	Bark	Phenolic compounds	Ethanol		IC_50_ (μg/mL)	Pine extract shows significant radical scavenging activity	[104]
				DPPH	111.40		
				H_2_O_2_	84.18		
				NO2	98.5		
*Thuja occidentalis*	Leaves	Flavonoids, phenols	Methanol	DPPH, FRAP	20–100 μg/mL	Crude extract shows significant antioxidant activity	[152]
*T. occidentalis*	Non-woody branches with leaves	Polyphenol, flavonoids	Mother tincture (MT)	DPPH, ORAC, NO	25 or 50 mg/kg	*T. occidentalis* mother tincture displayed 88.3% antioxidant activity by DPPH and about 78% by NO assay	[126]
*Taxus baccata*	Leaves and cones	Flavonoids, phenols	Methanol	DPPH	IC_50_ (μg/mL) 105.41, 518.51 leaves and cones resp.	Acetone and ethyl acetate extract of leaves show good scavenging activity	[153]
			Water	DPPH	533.66, >1000 leaves and cones resp.		
			Acetone	DPPH	25.24, 81.43 leaves and cones resp.		
			Ethyl acetate	DPPH	29.84, 180.26 leaves and cones resp.		
			Petroleum ether	DPPH	438.92, > 1000 leaves and cones resp.		
*T. wallichiana*	Leaves	Terpenoids, flavonoids			IC_50_ values (μg/mL)	The maximum DPPH activity was observed in methanol extract (91.25%), followed by water (87.64%), ethanol (85.23%), and ethyl acetate (83.27%) at the highest concentration (700μg/ml)	[154]
			Methanol	Superoxide radical	170.30		
				DPPH	212.00		
				LPO	126.09		
				Hydroxyl radical	82.34		
			Ethyl acetate	Superoxide radical	297.55		
				DPPH	301.80		
				LPO	151.96		
				Hydroxyl radical	199.05		
			Water	Superoxide radical	257.00		
				DPPH	258.29		
				Hydroxyl radical	175.33		
*T. wallichiana*	Leaf, stem	Polyphenols, flavanoids, terpenoids	Methanol	DPPH FRAP	IC_50_ value (μg/mL.) Leaves (23.18) Stem (56.75)	DPPH and FRAP activity of TW leaves and stem extract have high antioxidant activities.	[155]

PB-Pine bark; TW-Taxus wallichiana.

**Table 3:** Anti-inflammatory capacity of different conifers spp.

Conifer spp.	Part Used	Nature of Extract	Compounds	Major Method(s) of Testing	Dose. Conc	Main Effect	References
*Abies chensiensis*	Twigs and leaves	Ethanol	Terpenoids	Induce lipopolysaccharide to produce inflammation in RAW 264.7 macrophage cells	0.2–50.0 μM	4 compounds—3α-hydroxyl-8,14,22Z,24-tetraenlanosta-26,23-olide; (5*R*,20*R*)-8(14→13*R*)-abeo-17,13-friedo-3-oxolanosta-8,14(30),22*Z*,24-tetraen-26,23-olide; 8,14,22*Z*,24-tetraen-3-oxolanosta-26,23-olide; and (23*R*, 25*R*)-3,4-seco-9β *H*-lanosta-4 (28),7-dien-16α-hydroxyl-26,23-olid-3-oate—extracted from extracts showed significant anti-inflammatory activities of inhibition against NO formation with IC_50_ value of 15.9, 18.7, 20.18, and 10.9 μM	[125]
*A. georgei*	Aerial parts	Chloroform, ethyl acetate, *n*-butanol	Flavanoids	dimethylbenzene-induced ear oedema in mice	200 mg/kg	AG ethyl acetate extract shows 18% inhibition against dimethylbenzene-induced ear edema in mice while carrageenin-induced paw edema in rats shows inhibition ratios 28.2% and 35.6%, after 2 and 6h, respectively.	[156]
				Carrageenin-induced paw oedema rat	140 mg/kg		
*A. webbiana*	Leaves	Methanol/Petroleum ether extract	Flavanoids	Carrageenan-induced rat hind paw edema model in Albino mice	400 mg/kg	Plant leaves extract possesses significant anti-inflammatory properties	[157]
*Agathis robusta*	Leaves	Methanol	Flavanoids, tannins and saponins	Heat induced hemolytic method in human red blood cell (HRBC) membrane	400 μg/kg	Leaves extract shows good antiinflammatory activity	[158]
*Cedrus deodara*	Stem bark	Methanol	Deodarin, quercetin, taxifolin	Carrageenin-induced paw edema in Albino rat	100 mg/kg	Anti-inflammatory activity with 43.47% inhibition	[159]
*Cupressus macrocarpa*	Leaves	Methanol	Cupressuflavone (CUF)	Carrageenan-induced paw edema model in Mice	40, 80, and 160 mL/kg	CUF demonstrated antiinflammatory activity by inhibiting paw edema with 55, 60, and 64%, by decreasing the plasma pro-inflammatory mediators PGE2, IL-6, TNF-a and IL-1b	[46]
*Juniperus communis*	Berry	Alcohol/Water	Flavanoids (quercetin rutin, apigenin) chlorogenic acid	Acute-dextran and kaolin subacute inflammation induced in Wistar Rat	10 mL/kg	The antiinflammatory action of the juniper extract, administered as a microemulsion in acute-dextran model was increased when compared to kaolin subacute inflammation induced model.	[42]
*J. oxycedrus*	Berry	Ethanol, n-butanol	Flavonoids (amentoflavone, cupressuflavone, hinokiflavone, and rutin)	Carrageenan-induced hind paw edema model in mice	100 mg/kg	Ethanol extract of Joso berries displayed remarkable inflammatory inhibition ranging between 24.5% and 23.7% at 100 mg/kg in carrageenan-induced edema model	[160]
*J. foetidissima*	Berry	Ethanol	Flavonoids (amentoflavone, cupressuflavone, hinokiflavone, and rutin)	carrageenan-induced hind paw edema model in mice	100 mg/kg	JFB extract at a dose of 100 mg/kg. shows high antiinflammatory effect 26.9%	[160]
*Pinus gerardiana, P. roxburghii, P. wallichiana*	Bark	Ethanol	Flavanoid, tannin	against albumin denaturation, HRBC membrane stabilization assay	2500 μg/mL	*P. roxburghii* extract showed highest (%) of inhibition and protection i.e 86.54 and 89.92 against albumin denaturation and HRBC membrane stabilization. However, *P. wallichiana* have least inhibition and protection percentage, i.e., 76.54 and 81.2%	[104]
*Taxus baccata*	Aerial parts	Methanol	Terpenoids	ear edema induced in mice	3.2 mg/ear	*T. baccata* extract displayed best activity	[21]
*T. baccata*	Bark	Ethanol	Alkaloids, terpenoids, flavonoids	carrageenan-induced paw edema in Wistar Albino rat	200 mg/kg	Percentage of inhibition is 44% at a dose of 200 mg/kg	[161]
*T. baccata*	Heart wood	Ethanol	Taxoids, lignans	carrageenan-induced hind paw edema model inS wiss albino mice	30–100 mg/kg	TBW shows significant antinociceptive and anti-inflammatory activities	[29]
*T. wallichiana*	Bark	Methanol	Tasumatrol B, 1,13-diacetyl-10-deacetylbaccatin III (10-DAD) and 4-deacetylbaccatin III (4-DAB)	carrageenan-induced paw edema and Cotton-pellet oedema model in Wistar rats and Swiss albino mice	20 and 40 mg/kg; 40 mg/kg	In a carrageenan-induced inflammation model, tasumatrol B at a dose of 20 mg/kg showed significant activity, while in a cotton-pellet edema model tasumatrol B was found to be highly significant at the dose of 40 mg/kg.	[22]
*Thuja occidentalis*	Non-woody branches with leaves	Mother tincture (MT)	Polyphenols, flavonoids	Administered 2,4,6-trinitrobenzenesulfonic acid to induce intrarectal colitis in mice	25 or 50 mg/kg	MT manage to relieve intestinal inflammation experimentally induce by TNBS in 7 days.	[126]

JFB—Juniper foetidissima berry; AG—Abies georgei; TBW—*Taxus baccata* heart wood.

**Table 4:** In vivo and in vitro anticancer and cytotoxic studies of conifer extracts.

Conifer spp.	Part Used	Nature of Extract	Compounds	In Vitro and in Vivo Model	Dose. Conc	Main Effects	References
*Abies georgei*	Aerial parts	Chloroform, ethyl acetate, *n*-butanol	Flavanoids	Human tumor cell lines-A549, QGY-7703, LOVO, 6T-CEM	77.5, 11.1, 7.8, 32.8 μg/mL	AGC extract has potent tumour and antiproliferative effects in humor tumor cell lines	[156]
				(Mice) S180 tumours cell lines	100, 200 and 400 mg/kg	AGC also exhibited activity in tumour growth inhibition in a dose-dependent manner, with ratios of 46.7, 53.1 and 31.0% at doses of 100, 200 and 400 mg/kg, respectively	
*Araucaria angustifolia*	Female strobili	Water	Fatty acids and polyphenols	Laryngeal carcinoma HEp-2 cells	100–500 μg/mL	AAE inhibit the activity of mitochondria complex I and induce redox stress and cytochrome c, which leads cleavage of nuclear proteins of larynx HEp-2 cancer cells	[162]
*Cedrus deodara*	Stem wood	Chloroform	Lignans (Matairesinol, dibenzylbutyrolactol, (−)-Wikstromol)	In vitro human cell lines (cervix, breast, colon, liver, CNS, prostrate)	In vitro cytotoxicity IC _50_ value-Wikstromol (71.31–93.63) and Matairesinol (50.84–95.36) μg/mL	CD lignin mixture have potent to show a cytotoxic effect at the maximum in CNS and at the minimum in liver against cancer cell lines in a dose-dependent manner at 100 μg/mL from 49 to 95%.	[34]
				Human T lymphoblast, acute lymphoblastic leukemia cell line, Molt-4 and human promyelocytic leukemia cell line (HL-60)	IC_50_ (μg/mL) 15	AP9-cd-induced endogenous NO production leads to the generation of peroxide and disruption of mitochondrial membrane potential, leading to apoptotic pathway activation Increase in sub-G0 fraction from 35 to 60% in 24 to 48h	[163]
				In vivo swiss albino mice (K562 cells)		The lignin mixture displays anti-cancer effects by regulating annexin V binding, intracellular caspase activities and DNA fragmentation	
*C. deodara*	Needle	Ethanol	Kaempferol, myricetin, isorhamnetin and quercetin	HepG2 cells	IC_50_ 114.12 μg/mL	TFPNCD shows potent cytotoxicity by inhibiting the growth of HepG2 cells in a dose-dependent manner Regulates cell cycle and apoptosis	[164]
*Cryptomeria japonica*	Leaves	Methanol	Flavonoids	Albino mice of Ehrlich Ascites Carcinoma (EAC).	100–400 μg/gm	Tumor cell count as well as the amounts of ascetic tumour cells in packed cells were significantly reduced in infected mice treated with MC	[165]
*Juniper communis*	Berry	Methanol and water	Phenolic compounds	CaCo2 and HeLa carcinoma cell lines	IC_50_ 1300–2500 μg/mL	Methanol and water extracts of JCB show potent antiproliferative activity against cancer cell lines	[166]
*J. taxifolia*	Leaves	Chloroform	Polyphenols and lignan	human leukemia (HL-60) cells	2.5 μg/mL	7α-hydroxysandaracopimaric acid, a diterpenoid compound obtained from *J. taxifolia* leaves, shows antitumor effects on HL-60 cells	[24]
*J. phoenicea*	Aerial parts	Chloroform	Polyphenols		IC_50_ values (μg/mL)	It is found that JPCF disrupts cell cycle progression in the G0/G1phase and shows apoptotic, antiproliferative and necrotic effects on cancer cells lines	[20]
				Human lung (A549)	34.2		
				Breast (MCF-7)	24.5		
				Liver (HepG2) cancer cells	57.6		
*J. foetidissima*	Needle	Methanol	Quercetin, rutin	Rat brain tumor (C6) cell lines	IC_50_ values (μg/mL) 10.65	*J. foetidissima* needle extract showed significant antiproliferative activity	[50]
*M. glyptostroboides*	Leaf	Water	Polyphenols	PC12 cells	25 μg/mL	*M. glyptostroboides* leaf extract shows a cytotoxic effect and prevents oxidative damage of neuronal PC12 cells, protecting them from apoptosis; it was also found to significantly inhibit the release of LDH, which may result from apoptosis or necrosis	[49]
*Picea wilsonii*	Whole plant	DMSO	ND	Human keratinocyte HaCaT cell lines	1–3 g/mL	PwM extracts inhibit the production of MCP-1 IL-6, IL-13 and but do not inhibit IL-8 production	[115]
*Pinus kesiya*	Woody twig	Ethanol	Phenolic compounds and flavonoids	Human hepatocarcinoma (HepG2) cell lines	IC_50_ (μg/mL) 52.0	PK Extract exhibited a potent cytotoxic effect in the HepG2 cell line	[167]
*P. kesiya*	Branch	Ethanol	Phenolic compounds and flavonoids	Human leukemic U937 cancer cells	IC_50_: 299 μg/mL	PK ethanol extract possesses anticancer activity against U937 human leukemic cells via apoptosis	[168]
*P. merkusii*	Leaves	Methanol	Phenolic compounds	MCF-7, A549, HT 1080 and HepG2 Huh-7 cancer cell lines	IC_50_ (μg/mL) 4.5, 16, 4.1, 5.6, 9.5	PM methanol extract possesses anticancer activity against human cancer cell lines	[169]
*T. baccata*	Leaves, cones	Methanol	Phenolic compounds	HCT-116 human colon cancer and MDA-MB-231 human breast cancer cell lines	IC_50_ μg/mL Leaves: 14.43 and 4.59 cones: 49.69 and 133.53	Methanol extracts of leaves had better activity on HCT-116 cells than seed cone extract, with IC_50_ values of 14.3 for 24 h and 4.59 for 72 h. Meanwhile, extracts did not show any significant cytotoxic effects on the cancer cell lines	[153]
*T. wallichiana*	Heartwood	Methanol	Lignans 1 (taxiresinol 1) 2, 3	colon, ovarian liver, and breast cancer cell lines	IC_90_ lignan 2 and 3 μg/mL Caco 2:0.08 and 0.056 and 0.251	Taxiresinol 1 shows anticancer activity against ovary, colon, liver and breast cancer cell lines, while lignans 2 and 3 were found to be most active against Caco-2 cell lines	[170]
*T. yunnanensis*	All parts	ND	α-Conidendrin	MCF-7 andMDA-MB-231 cancer cell lines	40 μM	α-conidendrin have the potential to inhibit human breast cancer cell lines MDA-MB-231 and MCF-7, showing viability of 73 and 82%, respectively	[31]
*P. roxburghii*	Leaves	Water and ethanol	Phenolic compounds	A549 human lung cancer cell line	111.2 and 112.7 μg/mL	PRL extract shows potent anticancer activity against cancer cell lines.	[171]
*Taxus cuspidata*	Branches and leaves	Water	Polysaccharides	MCF7	IC_50_ μg/mL	Purified polysaccharides (Pe4) on HeLa cells had the highest inhibitory effect, and its IC_50_ value is 89.9, while (Pe1) shows the best cytotoxic capacity against cancer lines HepG2 and MCF7, with IC_50_ conc. 132.0 and 169.0 μg/mL, respectively	[172]
					169.0		
				Hela	89.9		
				HepG2	132.0		
*Thuja occidentalis*	Leaves and non-woody branches	Mother tincture (MT)	Polyphenols including flavonoids	Caco-2 cells	25 or 50 mg/kg	Caco-2 cells exposed to H_2_O_2_ and *T. occidentalis* MT proves its radical scavenging activity by reducing GSH level by 103% and 98% as compared to TNBS group; MT also managed to reduce the lipid peroxidation	[126]
*T. occidentalis*	Leaves	Ethanol	ND	Human NSCLC (A549) cell lines	IC_50_ μg/mL	Extract of TO shows both anticancer and antiproliferative activities against NSCLC (A549) cell lines in a dose-dependent manner.	[173]
					282		
				Human normal embryonic cell lines (L-132)	376		
*T. occidentalis*	ND	Mother tincture (MT) Thujone-rich fraction (TRF)	Thujone	A375 human malignant melanoma cell line	200 μg/mL	TRF as compared with TO MT on exposure to A375 cells exhibited highly cytotoxic, apoptotic and antiproliferative effects, but TRF shows a lower growth inhibitory response towards peripheral blood mononuclear cell (normal cells)	[174]

ND—Not determined; AGC—*Abies georgei* chloroform extract; AAE—*Araucaria angustifolia* water extract; TFPNCD—total flavonoids from the pine needles of *Cedrus deodara*; PRL—*P. roxburghii* leaves.

#### 4.2.4. Neurodegenerative Diseases

Neurodegenerative diseases (NDs) are more common among the elderly and may even lead to death, and so are a major threat in the 21st century [175]. AD, Parkinson’s disease (PD), Huntington’s disease (HD), amyotrophic lateral sclerosis (ALS), frontotemporal dementia, and the spinocerebellar ataxias are examples of ND [176], whose main features include nitrosative/oxidative stress, mitochondrial dysfunction, aggregated proteins accumulation, synapse loss, neuro-inflammation and decreased neuronal survival [177]. The progression of ND is also affected by genetic and surrounding ecological factors [175]. Indeed, it has been stated that the appropriate mechanism behind the cause of ND is mitochondrial dynamics variation, which elevates the oxidative damage, altering the biological activity of respiratory complexes, which results in brain energy dysfunction [178]. These stimuli trigger cellular stress, which leads to the synthesis and release of brain-derived neurotrophic factor (BDNF), as well as the activation of transcription factor CREB (cAMP response element-binding protein), with consequent expression of Arc (synaptic plasticity), PGC-1 (cellular energy metabolism), and APE1 (DNA repair enzyme), as well as the activation of the tropomyosin-related kinase (Trk B) receptor family and other downstream protein kinases [179].

Neurotrophins avoid neuron degeneration by binding to and activating the Trk receptor family, which is located in the plasma membrane [177]. Neurotrophins work by binding to and activating the Trk receptor family, which is found in the plasma membrane, to prevent neuron degeneration. Since neurotrosphins bind to Trk receptors, they create a microenvironment that promotes neuron development [180]. Various intracellular signaling pathways, such as ERK and PI3k/AKT, are regulated as a result of this binding, allowing cells to survive and aiding in the recovery of neurons from neurodegeneration. Additionally to signaling pathway activation, neurotrophins support Bcl-2 gene expression, which inhibits intracellular apoptosis [180]. Thus, early diagnosis of neurodegeneration may allow for early treatment, which may help to prevent the disease from progressing further [181]. Inhibition of the N-methyl-D-aspartate (NMDA) receptor can prevent or postpone AD. The drugs memantine and namzaric, which act as antagonists for the NMDA receptor, are used to treat AD patients [182].

Bioactive molecules have been recognized for their valuable biological effects, including neuroprotective properties, such as the ability to regulate mitochondria in a way that is distinct from TMs [183]. Branco et al. (2018) found that the flavonoid-rich *A. angustifolia* bracts extract (AAE) has neuroprotective properties by restoring rotenone-induced mitochondrial complex I, inhibiting the formation of lipid peroxidation and neuronal ROS, and through over expression of NDUFS7 protein and NDUFV2 gene levels in human dopaminergic SH-SY5Y cells [43]. Bark extract of *P. pinaster* shows protective effects against oxidative hemolysis induced by H_2_O_2_, the formation of thiobarbituric acid reactive products and lipid peroxidation [184]. In addition, it prevents oxidative damage to many proteins aggregation and may lessen the risk of several NDs, such as AD, PD and HD [185]. The neuro-protective potential of various conifer spp. crude extracts is summarized in Table 5.

#### 4.2.5. Alzheimer’s Disease (AD)

AD is a common neurodegenerative disease that affects 80% of the elderly population, accounting for about half of all dementia cases and ultimately results in death [186]. Its symptoms include failure to learn, gradual memory loss, and deterioration in behavior and neuronal function [187]. Regarding treatment, only five approved treatment options are licensed in the European Union for the treatment of AD, including rivastigmine, donepezil (cholinesterase inhibitors (ChEIs), galantamine, and memantine (NMDAR antagonist) [188]. An antimitotic agent paclitaxel widely used for the treatment of lung, ovarian and breast cancer has also been investigated as a possible treatment for AD [189]. It is mainly effective in the treatment of tauopathies, which are disorders caused by mutations in the tau protein, which is abundant in central nervous system (CNS) cells and acts by stabilizing microtubules [190]. The consumption of polyphenol-rich foods or beverages has been related to the prevention of AD in distinct studies [191]. The accumulation of amyloid-(A) in brain and leptomeningeal vessels causes cerebral amyloid angiopathy (CAA), which is also a central component of neuritic plaques in AD amyloid-(A) and has been related to the pathogenesis of two of the most common forms of dementia: AD and CAA. As a result, Aβ should be a top priority in the treatment of these diseases, which currently have no effective therapies [192]. Taxifolin, an antioxidant and anti-glycation flavonoid, reduces Aβ aggregation and its accumulation in the cerebrovascular system. In vitro studies have shown that taxifolin facilitates Aβ clearance in the brain, prevents Aβ fibril formation and CAA cognitive loss, and increases cerebral blood flow [193]. The methanol extract of *P. roxburghii* bark contains bioactive compounds, such as quercetin and gallic acid, which play important roles in neuroprotection by reversing mitochondrial dysfunction, free radical formation, and improving memory and cognition in rats, as well as reducing oxidative stress by improving acetylcholine levels. Furthermore, anti-AD activity has been documented in *Pinus* species, such as *P. halepensis* and *P. massoniana* [194]. Piceatannol, a compound derived from pine bark, has proven to be effective in preventing AD [195]. Resveratrol (RV), a stilbenoid, protects neurons from oxidative damage in a variety of ways, such as lowering lipid peroxidation and increasing intracellular antioxidant levels including antioxidant enzymes catalase (CAT), superoxide dismutase (SOD), glutathione peroxidase (GPx), and heme oxygenase 1 (HO-1) [196]. In this way, RV acts as an anti-AD agent by reducing neuroinflammation, inhibiting Aβ-plaque formation and tauopathy, and as a result inhibits neuronal death and improves memory [197]. Pycnogenol derived from *P. pinaster* bark has antioxidant, anti-inflammatory, and neuroprotective properties, including inhibition of amyloid-induced neuron apoptosis [198]. When the effect of pycnogenol was investigated in AD-related pathology in a β-amyloidosis mouse model, a decline in plaque numbers was found, while no changes were reported in the soluble β-amyloidosis levels, astrocytes, neurons, microglia, myelination pattern, morphology of axons and the gene expression of APP-processing enzymes [199]. Hence, it is suggested that pycnogenol has potential use in the prevention or in early stages of AD and mild cognitive impairment (MCI) [200]. Table 5 summarizes the neuroprotective potential of different conifers’ phytochemicals in AD.

#### 4.2.6. Parkinson’s Disease

PD is second to AD in terms of the most prevalent progressive ND, with an estimated global prevalence of over 10,000,000 cases [201]. The selective loss of dopaminergic neurons in the substantia nigra pars compacta (SNpc) leads to PD. Briefly, PD occurs due to oxidative stress, dysfunction of mitochondrial complex-1, oxidative cell damage, neuronal excitotoxicity, calcium homeostasis, apoptosis, distressed energy metabolism, inflammation and protein aggregation, such as a-synuclein, apoptosis, and interaction between genetic and environmental causes [202]. Due to uncoordinated mouth and throat movements, PD causes bradykinesia, muscle rigidity, rest tremor, and the loss of postural control, as well as certain secondary symptoms, such as dementia, sialorrhea, soft voice, and trouble swallowing [203,204]. Oxidative stress generates ROS that causes oxidative damage, such as 4-hydroxynonenal (HNE), 26S proteasome and interferes with dopamine metabolism leading to PD [85]. Changes in protein ubiquitination and degradation have recently been related to dopaminergic cell death in PD [205]. Presynaptic protein α-synuclein (α-syn) influences the release of neurotransmitters from synaptic vesicles in the brain [206]. Currently, the treatment of PD includes drugs such as L-DOPA, which is catalyzed primarily by dopa decarboxylase in the brain, and some others such as ropinirole, selegiline, and rasagiline. Ropinirole has some adverse effects, including ankle oedema, vomiting, nausea, hypotension, insomnia, weight loss, hallucinations, psychosis, arrhythmia, dry mouth, nightmares, persistent diarrhea, somnolence and constipation, limiting their clinical applications [207]. As a result, the focus of rising interest in alternative treatments for ND, such as PD, has turned to natural products, which can provide alternatives due to their high effectiveness and few side effects [208]. Many plant extracts tend to stop α-syn from oligomerization and fibrillation, which is an emerging therapeutic mechanism in PD [183]. Methanol extract of *J. communis* at doses of 100 and 200 mg/kg was found to be effective in reducing catalepsy, enhancing locomotor activity (actophotometer), and increasing the level of reduced glutathione (GSH), protein level and muscle activity in rats [209]. In an in vitro study on Fisher F344 rats, Zhang et al. (2010) discovered that RV protect dopaminergic neurons from damage caused by MPP+, 6-OHDA, and also show efficacy against lipopolysaccharide-induced neurotoxicity by inhibiting nuclear factor kappa B (NF-κB) signaling and microglial activation [210].

#### 4.2.7. Insomnia

Insomnia is a chronically debilitating disease that has become increasingly common, posing immense health and economic challenges for both individuals and the community [211]. Trouble falling asleep, staying asleep, fragmented sleep (repeatedly waking up at night or waking up early in the morning) are all symptoms of this condition [212]. While behavioral therapy, psychotherapy and light therapy have all been used to treat insomnia, the most common medications for insomnia are hypnotic drugs that target GABAA-benzodiazepine (BZD) receptors, such as diazepam and zolpidem [213]. However, several side effects have been identified, including cognitive impairment, resistance, headaches, nausea, and rebound insomnia [214,215]. Methanol extract of *A. webbiana* leaves showed potent synergistic effect in mice at dose of 100, 150, and 200 mg/kg, with sleep-inducing sedative drugs, diazepam (6 mg/kg), pentobarbitone sodium (50 mg/kg) and propylene glycol [157]. In addition, the major monoterpenoid components present in *Pinus* spp., α-pinene and 3-carene, have been reported to have hypnotic effects through GABAA-BZD receptors. 3-carene increases the length of sleep in mice given pentobarbital-induced sleep drugs by binding to the BZD site of the GABAA-BZD receptor α1 and ϒ2 [216].

## 5. Other Activities

### 5.1. Antidiabetic Activity

Diabetes mellitus is one of the world’s most serious health issues, with a rising prevalence and mortality rate [217]. Insufficiency in blood sugar control has significant health implications. Anti-diabetic medications are successful, but they come with unwanted side effects. Medicinal plants, on the other hand, can act as an additional reservoir of anti-diabetic agents [218]. Insulin and synthetic oral drugs hypoglycemic drugs are the most commonly used treatments for diabetes, despite the fact that they do not fully reverse the disease’s complications and have severe side effects. This is the driving force behind the search for new anti-diabetic agents [219]. After six years of treatment, sulfonylureas are expected to lose effectiveness in 44% of patients, while glucose-lowering drugs have been stated to be unable to control hyperlipidemia [220]. Nonetheless, the quest for newer antidiabetic drugs from natural sources continues due to many drawbacks associated with the use of current synthetic antidiabetic drugs [221].

Many plants have long been known to be a significant source of effective antidiabetic drugs in developing countries, especially to reduce the cost of conventional treatments [217]. Phytoconstituents, such as terpenoids, flavonoids, alkaloids, carotenoids, saponins, glycosides, which have antidiabetic effects, are now used to treat diseases such as diabetes [219,222]. Indeed, the number of people living with diabetes is rising, stoking concerns among medical professionals and the public. Despite the availability of antidiabetic medications in the market, medicinal plants are also effective [217]. The *Araucaria, Cedrus, Juniperus, Pinus, Thuja*, and *Taxus* genera have all been studied for their antidiabetic, antihyperglycemic, and hypoglycemic properties, as well as their ability to inhibit α-amylase and α-glucosidase and shown in Table 6.

In vitro experiments exhibited that the ethanol extract of *P. halepensis* bark had a greater inhibitory effect on the enzymes involved in diabetes (α-amylase and α-glucosidase) with IC_50_ values of 234.26 and 7.97 µg/mL, respectively [151]. Piceatannol is a phytochemical that has antidiabetic properties. Piceatannol, a resveratrol analogue, restores palmitic acid-induced disruption of insulin signaling and endothelial NO production in human endothelial cells by activating anti-inflammatory and antioxidant mechanisms (HO-1) [223]. According to Vallianou et al. (2013), the antihyperglycemic property of resveratrol appears by increasing the glucose transporter activity that occurs in the plasma membrane; the results indicate that the key antihyperglycemic action effects of resveratrol are due to the SIRT1 activation and AMPK (5’ AMP-activated protein kinase) [224]. RV antidiabetic activity is linked to its ability to increase AMPK and SIRT1 expression/activity in different tissues of diabetic subjects [225]. The multi-target effects of RV against diabetes were well-defined by Bagul and Banerjee, 2015, who underlined an improvement in insulin sensitivity and GLUT-4 translocation, while oxidative stress was reduced, carbohydrate-metabolizing enzymes were regulated, SIRT1 and AMPK were activated, and adipogenic genes were decreased [226]. As a result, lowering glucose levels by inhibiting enzyme activity is an effective method for treating hyperglycemia through using natural products.

### 5.2. Anticonvulsant Activity

Epilepsy is a neurological condition that affects people of all ages all over the world. The side effects of antiepileptic drugs and their connection to oxidative stress have prompted researchers to look for new medications that are less expensive and that have fewer side effects [227]. Several natural compounds derived from various conifer species have shown good anticonvulsant properties in animal models [228]. In India, extracts of *C. deodara* wood and *P. roxburghii* bark have historically been used to treat neurological disorders. In this analysis, the anticonvulsant activity of 3,4-bis(3,4-dimethoxyphenyl)furan-2,5-dione (BDFD) isolated from the ethanol extract of *C. deodara* and quercetin, chlorogenic acid, and rutin isolated from the ethanol extract of *P. roxburghii* bark were assessed in mice, and the results demonstrate modulation in the function of glutamate receptors by enhancing inhibitory GABA minergic neurotransmission [228,229]. Hinokiol, a neuromodulatory compound isolated from *Taiwania cryptomerioides*, affects NG108-15 cells and rat hippocampal CA1 neurons or neuronal ion channel activities by inhibiting voltage-gated Na(+) channels (VGSC) [230]. Lectins, normally a glycoprotein extracted from seed of *A. angustifolia*, had an antiseizure effect in strychnine and pentylenetetrazole-induced seizure models, revealing positive effects in the activation of glycinergic and GABAergic systems, respectively, and caused a reduction in animal movements [231].

### 5.3. Analgesic Activity

Analgesia/pain is an intense, ill-defined feeling triggered by a stimulus (external/internal); it is the most significant symptom that serves as an alarm signal and is mainly defensive in nature [232]. Bradykinin, tumor necrosis factor (TNF), and ILs cause analgesia by blocking the pain nerve sensitizing pathway [233]. An analgesic is a drug that relieves pain by acting on pain mechanisms in the CNS or in the peripheral nervous system (PNS) without affecting consciousness [234]. Even after new advances in pain therapies, healthcare professionals still need safe, reliable, and effective analgesic drugs to treat a variety of painful conditions, especially chronic pain. Based on its traditional medicinal uses, isolated *T. wallichiana* constituents are widely explored for analgesic purposes [22]. Indeed, the analgesic activity of *C. deodara* methanol bark extract was observed in Albino rats with acetic acid-induced writhing and found that it had a major analgesic effect, with 55.8% defense at a dose of 100 mg/kg [159]. In the acetic acid-induced writhing and hot plate model, *Cupressus* flavanone (CUF) demonstrated significant analgesic activity. At the three CUF doses used, 160 mg/kg in 120 min prevented PG synthesis and writhing response in mice at a rate of 25, 48, and 62%, respectively [46].

### 5.4. Antinociceptive Activity

Heartwood ethanol extract of *T. baccata* taxoids and lignin derivative compounds exhibited potent antinociceptive activity against p-benzoquinone-induced abdominal contractions in mice [29].

### 5.5. Antimicrobial Activity

Coniferous tree extracts are attracting intensified interest among scientific communities due to their possible applications in food, medicine, and cosmetics. Among conifers spp., various extracts have recently been identified as a significant source of bioactives with antimicrobial potential, as shown in Supplementary Materials Table S1.

**Table 5:** Conifers’ phytochemicals demonstrating neuroprotective potential in vitro and in vivo.

Conifers spp.	Compounds with Neuroprotective Potential	Model	Effective Concentration	Relevant Bioactivities	Reference
*Abies holophylla*	Holophyllin-D	C6 glioma cells	20 μM	Diterpenes compound holophyllin D shows neuroprotective potential in C6 glioma cells by inducing nerve growth factor	[25]
*Araucara angustifolia*	Catechin, epicatechin and rutin	Rat	10 mg/mL	AAE has antioxidant and neuroprotective properties as it decreases the TBARS levels, CAT activity and NO production in the hippocampus region of the brain in rats.	[235]
*A. angustifolia*	Catechin, epicatechin, rutin, quercetin and apigenin	human dopaminergic SH-SY5Y cells	5 μg/mL	Decrease in the production of neuron (ROS) and lipid peroxidation.	[43]
*A. angustifolia*	Quercetin	cockroach	200–400 μg/g	Neurotoxicity modulates the behavior of insects by altering the dopaminergic pathways, as quercetin has the ability to induce selective inhibitory actions on NMDA and GABA receptors and inhibit the enzyme acetylcholinesterase (AChE)	[236]
*Cedrus deodara*	Cedrin	PC12 cells	0.1, 1 and 10 μM	PC12 cells injured by amyloid β1–42 can be improved by cedrin. Cedrin can reduce (ROS) overproduction, enhance the activity of SOD and decrease MDA content and inhibition of oxidative stress, improvement of mitochondrial dysfunction and suppression of apoptosis in PC12 cells	[237]
Metasequoia glyptostroboides	Gallic acid, rutin, myricetin, kaempferol, quercitrin, epigallocatechin, epicatechin gallate epigallocatechin gallate and caffeic acid	Neuronal PC12 cells	2 mg/mL	The extracts effectively reduced the hydrogen peroxide-induced lipid peroxidation in neuronal PC12 cells by decreasing intracellular ROS accumulation	[49]
*Pinus densiflora*	Catechin, quercetin dehydrate, astragalin and kaempferol	Mice	50–100 mg/kg	Catechin displayed a potential effect protecting mouse brains from oxidative damage via the improvement of the antioxidant capacities of TAC, the GSH-redox system, SOD and CAT in the hippocampus region as well as the inactivation of cytokines such as NF-kB in pyramidal cells of the hippocampal CA1 region, while PNE shows antiamnesic properties and effects in Alzheimer’s, as it attenuated the increase in serum corticosterone level and up-regulation of GR hippocampal gene expression	[238,239]
*P. eldarica*	Needle extract	Mice	50 mg/kg	Alkanes, sterols, terpenoids, and quercetin, which is found in *P. eldarica*, help in inducing sleep and alter the sleep–wake cycle partly via activation of GABA receptors	[240]
*P. massoniana*	Polyprenols	Mice	25 mg/kg	Polyprenols significantly increased T-AOC, GSHPx, damaging peroxide components from cells in order to stop the lipid peroxidation chain reaction and avoid excessive hydrolysis to form NEP, MDA, SOD activity (remove free radicals) and β-site AβPP cleaving enzyme 1 (BACE1) expression, while NOS activity, MDA concentration, NO, concentration of Aβ1-42 and PS1 were reduced	[241]
*P. pinaster*	Pycnogenol (PYC)	Mice	20 mg/kg	In the MPTP-induced mouse model, PYC could prevent dopaminergic neurons by reducing oxidative loads, suppressing glial cell activation, and inhibiting inflammatory responses	[100,242]
*P. roxburghii*	Quercetin, rutin, gallic acid	Wistar albino	100–300 mg/kg	Quercetin and gallic acid, both present in stem bark, have been shown to inhibit neuronal toxicity and apoptosis by reversing mitochondrial dysfunction and free radical development	[243]
*Thuja occidentalis*	Water extract	Mice	100 mg/kg	CNS depressant activity, anticonvulsant and muscle relaxant activity	[244]
*Torreya nucifera,*	Arctigenin	Rat Cortical cells	0.01 µM to 10.0 µM.	Arctigenin significantly attenuated glutamate-induced neurotoxicity by inhibiting the binding of [3H]-kainate to its receptors	[35]
*T. semen*	Polyphenols, flavonoids	Mice	0–10 mg/mL	TS increased the level of total glutathiones	[245]

T-AOC—total antioxidative capacity; GSHPx—glutathione peroxidise; SOD—super oxide dismutase; NEP—neprilysin; MDA—malondialdehyde; NO—nitric oxide, NOS—nitric oxide synthase; PS1—presenilin 1, CAT—catalase.

**Table 6:** Antidiabetic activity of different conifer extracts.

Conifer spp.	Part Used	Compounds	Model	Induction of Diabetes	Dose. Conc	Effects	References
*Araucaria cunninghamii*	Seeds	Glucomannan	Albino wistar rats	Streptozotocin	25 and 50 mg/kg	Glucomannan reduce blood glucose level due to presence of D-glucosyl and β-1, 4-linked D-mannosyl units Decreases total cholesterol (TC) total glycerides (TG), high density lipoproteins cholesterol (HDL-C), very low-density lipoprotein cholesterol (VLDL-C) and low-density lipoprotein cholesterol (LDL-C)	[246]
*Cedrus deodara*	Heart wood	Flavonoids	Wistar albino rat	Alloxan	500 mg/kg	Reduction in blood sugar level from 49.79% within 21 days.	[247]
*Juniperus* *communis*	Berry	Flavonoids	Wistar rat	Streptozotocin	250 mg/kg	JCB extract have potential to increase the peripheral glucose absorption and plasma insulin levels	[248]
*J. communis*	NR	NR	Rat	Streptozotocin-nicotinamide	100–200 mg/kg	Reduction in blood glucose levels Antihyperlipidemic activity in the form of the reduction in TG TC, LDL, and VLDL dose-dependently	[249]
*J. oxycedrus*	Leaves	Linolenic acid, oleic acid	Wistar-albino rats	Streptozotocin	500 and 1000 mg/kg doses	Leaf extracts rich in unsaturated fatty acids responsible for activating PPAR ϒ receptors or increasing the release of insulin from beta cells of the pancreas to reduce glucose levels	[250]
*Pinus gerardiana*	Nut	Flavonoids	Rat	Alloxan	250, 500, and 750 mg/kg	Decrease the blood glucose level by inhibiting alpha-amylase enzyme activity Antihyperlipidemic	[251]
*P. halepensis*	Bark	Phenolic compounds	Rat	Glucose	250, 500 mg/kg	Stop enzymes (α-glucosidase and α-amylase) implicated in sugar metabolism antioxidant	[151]
*P. pinaster*	Bark	Phenolic compounds	NR		IC_50_ (µg/mL) at PB 70% and PB 50%	PBEs ethanol extract at PB.70% and 50% shows higher α-amylase and β-glucosidases inhibitory activity, respectively	[39]
				α-amylase	254.2		
				β-Glucosidase	122.7		
*P. pinaster*	Bark	Pynogenol	Human	NR	100 mg	Reduce blood glucose level in blood Elevated antioxidant defense mechanisms	[252]
*P. roxburghii*	Bark	Quercetin	NR	Alpha amylase inhibitory activity	100 μg/mL	Quercetin present in extract displayed significant enzyme inhibitory activity against α-amylase, with 49.6% inhibition.	[171]
*P. roxburghii*	Bark	Quercetin	Rat	Alloxan	100, 300 and 500 mg/kg	Anti-hyperglycemic activity of *P. roxburghii Sarg*. extract increase the releasing of beta cell regeneration against alloxan induced free radicals Antihyperlipidemic activity	[253]
*Taxus cuspidata*	Branches and leaves	Water	Polysaccharides Pe4 (arabinose, galactose, glucose, xylose, mannose)	NR	10–120 μg/mL	Pe4 showing good type 2 antidiabetic activity by inhibiting α-glucosidase Inhibit human cervical cancer	[172]

NR—Not reported.

Conifer compounds act as antimicrobials because they have potential in degrading microbial cell walls: disruption to the cytoplasmic membrane and membrane proteins, cell leakage, cytoplasm coagulation, and proton motive force depletion are all examples of their inhibitory action [254]. The following is a list in descending order of the key bioactive compounds responsible for antimicrobial effects: ketones > alcohols > esters > hydrocarbons > aldehydes > ketones > alcohols > esters > hydrocarbons [255]. Terpenoid compounds (α-terpineol, δ--3-carene, geranyl acetate, borneol, α and β-pinene, limonene, α-terpinene, ϒ-terpinene, β-ocimene, bornyl acetate, 1,8-cineole, α-phellandrene, p-cymene, linalool, ϒ-muurolene, α-humulene, and cadinene) have been found to be responsible for antimicrobial activity [8,33]. Alkaloids, especially 1,6-dehydropinidine obtained from *P. abies* needle and bark, have recently been discovered to have antimicrobial activity against *Streptococcus equi* (MIC = 55 g/mL) [13]. Secoisolariciresinol, pinoresinol, eudesmin, lariciresinol, and lariciresinol-4-methyl ether isolated from *A. araucana* wood methanol extract have shown potent antibacterial and antifungal activity with a synergistic effect, enhancing their potency against bacteria and fungi [256]. Anti-herpes activity was found in hydroethanolic extract ethyl acetate (EA) and n-butanol (NB) fractions from *A. angustifolia* leaves, indicating that conifer spp. could have been used in folk medicine to treat viruses [45]. It has been reported that RV, piceatannol, hydroxystilbenes and isorhapontigenin are present in debarking water, a byproduct of debarking logs of *P. abies*, meaning that it has the potential to prevent the growth of a variety of fungi and may be used as a natural fungicide [257].

### 5.6. Larvicidal Activity

In recent years, there has been increased interest in secondary metabolites with potential larvicidal activity in a number of countries around the world [258]. Dengue fever, yellow fever, dengue hemorrhagic fever, malaria and chikungunya are the most severe diseases transmitted by mosquitoes. *Aedes aegypti* is one of the mosquito species involved in the transmission of such vector disease outbreaks [258,259]. Larvicidal activity has been documented in extracts of conifer spp. parts [260]. The mosquito control technique is determined by the larval stages (egg, larvae, pupae, and adult) of the target. Mosquito control methods include spraying chemical insecticides on adult mosquitoes or destroying mosquito larvae before they grow into adults, either by means of synthetic larvicides or by using botanical extracts as an alternative larvicide [258]. The use of these synthetic insecticides against mosquitoes creates insecticide resistance as well as multifarious problems, such as environmental pollution and poisonous hazards to human beings [261]. These plant-oriented natural products are eco-friendly in nature and are preferred for use against larvae over other synthetic insecticides [262]. Based on mortality, the ethanol extract of *J. procera* and *T. orientalis* leaves has potential against *Anopheles arabiensis*, *A. stephensi* and *Culex quinquefasciatus* larvae [260,263]. It also has been found that *C. sempervirens* petroleum ether leaves extract shows a toxic effect on *Musca domestica* larva and also causes a decrease in the production of eggs and fecundity, as well as inducing sterility in both males and females [264]. Ethanol extract of *Pinus caribaea* and *P. merkusii* leaves and bark exhibited the highest mortality in the larvae of *A. aegypti*, a vector responsible for dengue fever transmission [265].

### 5.7. Cardiovascular Diseases

The rate of death due to cardiovascular diseases is quite high. Several medications are available to treat cardiovascular disorders and their complications. The general public has come to recognize the use of functional foods or dietary supplements to treat cardiovascular diseases [266]. A study reported that pycnogenol supplementation regulates the circulation in blood vessels, and reduces mild hypertension, cardiovascular diseases and platelet aggregation stimulated by smoking [267]. RV protects the heart by inhibiting platelet aggregation, thromboxane A2 formation (vasodilator effect), and Cox-1 peroxidase reactions [268]. In addition, low doses of RV (such as those present in the average diet) have been shown to have cardioprotective effects [269]. Cardiovascular disorders are common in both developing and developed countries. Piceatannol is intended to help to prevent cardiovascular disorders, including arrhythmia, high cholesterol, angiogenesis, and atherosclerosis [266]. Piceatannol pretreatment decreases cardiac hypertrophy, as measured by hypertrophy marker expression levels, cross-sectional area, and heart weight/body weight ratio. It also prevents lentiviral GATA-6-induced cardiac hypertrophy [270].

## 6. Clinical Trials

Clinical trials using extracts from conifer species in humans are limited. Only a few studies have reported the use of conifer spp. extracts in humans for inflammation and cardiovascular issues. A randomized 10-day, double-blind clinical trial was conducted on traumatic brain injury (TBI) patients. A pycnogenol supplement (OLIGOPIN) was orally administered with an oral dose of 150 mg per day, conducted in 60 people, with 30 control (Placebo) and 30 taking the PYC supplement, and it was found that PYC is effective in reducing inflammation and oxidative stress in TBI patients by increasing the level of pro-inflammatory cytokines, e.g., IL-6, TNF-α, IL-1β, and C-reactive protein (CRP) [271]. Another pilot study with a length of 12 weeks was conducted with Enzogenol^®^ at an oral dose of 480 mg/day in 26 healthy people aged between 55 and 75 years. Some significant results have been published, such as beneficial changes in anthropometric data, a reduction in unnecessary body fat, vascular and plasma rheological indices, with a reduction in blood pressure and cardiovascular-related problems [272]. Nowadays, many products such as supplements, gels, creams, lotions, capsules, tablets, ointments formed from conifers’ bark, and needle powder are sold commercially. Table 7 describes the main characteristics of these products.

**Table 7:** Conifer-derived commercially available products sold on the global market.

Plant	Part Used	Trade Name	Phytochemicals Composition	Formulation	Dose/Duration	Product
*Pinus pinaster*	Bark	Pycnogenol	Catechin, taxifolin, procyanidins, caffeic, p-hydroxybenzoic, ferulic, acids	Tablets, liquids, chewing gums, gels, ointments, capsules or lotions	150 mg/day for 6 months	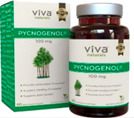
*P. pinaster*	Bark	Oligopin	Caffeic acid, catechin, epicatechin, taxifolin and ferulic acid	Capsules	150 mg for 10 days	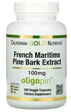
*P. radiata*	Bark	Enzogenol	Flavanoids, proanthocyanidins	Tablets	480–960 mg/day for 5–6 months	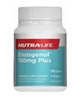
*Picea abies*	Needles	Ropren	Flavanoids	Tablets, capsules, lotions	8.6 mg/kg for 28 days	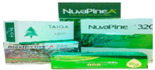
*Taxus brevifolia*	Bark	Taxol	Paclitaxel	Injections	30 mg/m^2^ every 3 weeks	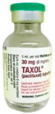
*P. massiona*	Bark	Not found	Polyphenols, flavanoids, proanthocyanidins	Capsules	1 capsule daily	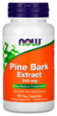

## 7. Phytotoxic Effects of Conifer Extract

Despite all of the advances stated so far, toxicity studies are required to know the effective doses that could be administered subsequently, as well as to depict the potential clinical signs elicited by the plant material [273]. Few toxicological studies have been reported on *T. baccata, P. contorta* and *J. communis* needles. A study was performed on a sample collected from a fatal case of a 22-year-old man, which revealed the presence of taxol A, diterpenoids such as monoacetyltaxine, and cardiotoxic compounds, such as 10-deacetylbaccatin III and taxine B. These compounds bind to calcium channels in cardiac myocytes, causing nausea, seizures, vomiting, dizziness, several cardiovascular effects, including bradycardia, and leading to ventricular tachycardia with severe ventricular arrhythmias, ventricular fibrillation and abdominal pain [274]. In another study, it was found that pine and juniper needles at an oral gavage dose of 62–245 mg/day in cattle have an abortifacient effect due to the presence of isocupressic acid in high doses [271]. Therefore, it is unsafe to feed pine and juniper needles to gravid cattle.

## 8. Conclusion and Future Trends

In this review, we have discussed the traditional and pharmacological uses of various conifers’ extracts against diabetes, neurological disorders, inflammation, and cancer. The phytochemical constituents present in conifer extracts are nontoxic at therapeutic levels, with polyphenolic compounds having significant biological activities. Stilbenes, terpenes, alkaloids, lignins and flavanoids, such as quercetin, rutin, resveratrol, and the compounds PYC and enzogenol, are the phytochemical components of conifer extracts reported to have sedative, antidiabetic, anticancer and anesthetic effects. In addition, phytochemicals present in conifer extracts assist in the regulation of glucose and lipid metabolism, insulin secretion, stimulating β cells, the NF-kB signaling pathway, the inhibition of gluconeogenic enzymes, ROS protective action as well as targeting and modulating cytokines which affect neuron cells and reduce oxidative stress. In this way, conifers’ phytochemicals are used as an alternative to synthetic drugs and can be to a greater extent in the future, as they can be helpful in the formulation of new drugs. Without a doubt, conifers’ phytochemicals are the natural sources of future drugs; in the field of drug discovery, a large number studies into phytochemicals are still required. More efforts are needed to investigate and assess the clinical potential and molecular characterization of medicinal compounds with the help of databases and interdisciplinary group efforts.

## Supplementary Materials

The following are available online, Table S1: Antiviral, antibacterial and antifungal activity of different conifers’ extracts.
