# Supplementary material for: Conifers Phytochemicals: A Valuable Forest with Therapeutic Potential
Source: Molecules. 2021 May 18;26(10):3005. doi: 10.3390/molecules26103005 (PMC8158490; doi:10.3390/molecules26103005)
Supplement: Supplementary file 1 [file molecules-26-03005-s001.zip › molecules-1210382-supplementary.pdf]

Table S1. Antiviral, antibacterial and antifungal activity of different conifers' extracts

Conifer spp.	Part Used	Nature of Extract	Compounds	*In vitro* and *in vivo* Model	Extract Conc./ MIC/MBC	Temp. and Duration	Main effects	References
Araucaria angustifolia	Leaves	Hydroethanolic extract	Biflavonoids	Vero cells lines	IC$_{50}$ 46.82 µg/mL	37°C, 2 h	hydroethanol extract n-butanol (NB1) fractions displayed best antiherpetic activity against Herpes Simplex Virus	[1]
A. cookii	Leaves	Methanol, chloroform, petroleum ether	Phenolic compounds	*Psudomonas* spp., *Klebshiela* spp.; *Aspergillus flavus, A. niger*	1000 µg/mL	37°C, 24h; 28°C, 24h	Extracts exhibited good activity against all bacterial spp. with inhibition zone 7 mm but do not displayed any activity against *Aspergillus* spp.	[2]
A. cunninghamii	Leaves	Methanol	Phenolic compounds	*Erwinia chrysanthemi*	125/250 µg/mL	37°C, 24h	Methanol extract have potential against all bacteria spp. as it shows inhibition range range activity from 54 % to 81%	[3]
				Bacillus subtilis	62.5/125 µg/mL	37°C, 24h		
				Escherichia coli	62.5/250 µg/mL	37°C, 24h		
				Xanthomonas phaseoli	31.25/125 µg/mL	37°C, 24h		
	Leaves	Ethanol	Phenolic compounds	*B. subtilis*	62.5/125 µg/mL	37°C, 24h	While ethanol extract inhibition range from 45-72%.	
				Agarobacterium tumefaciens	31.25/125 µg/mL	37°C, 24h		
				E. coli	62.5/250 µg/mL	37°C, 24h		
				X. phaseoli	125/500 µg/mL	37°C, 24h		
Biota oriantalis Endl	Leaves	Methanol	Phenolic compounds	*A. tumefaciens*	125/na µg/mL	37°C, 24h	Methanol extract shows potency against all the bacteria with activity range from 54 % to 81% however, ethanol extract activity ranges from 45-72%.	[3]
				B. subtilis	62.5/125 µg/mL	37°C, 24h		
				E. chrysanthemi	62.5/250 µg/mL	37°C, 24h		
				E. coli	NF	37°C, 24h		
				X. phaseoli	125/500 µg/mL	37°C, 24h		
		Ethanol	Phenolic compounds	*E. coli*	125/250 µg/mL	37°C, 24h		
				X. phaseoli	125/500 µg/mL	37°C, 24h		
Cedrus deodara	leaves	Ethanol	Phenolic compounds	*B. subtilis*	62.5/125 µg/mL	37°C, 24h	Ethanol extract shows activity ranges from 45-72%.	[3]
				A. tumefaciens	125/na µg/mL	37°C, 24h		

				E. chrysanthemi	125/250 µg/mL	37°C, 24h		
				X. phaseoli	250/na µg/mL	37°C, 24h		
				E. coli	62.5/125 µg/mL	37°C, 24h		
Cephalotaxus griffithi	leaves	Methanol	Phenolic compounds	*A. tumefaciens*	62.5/125 µg/mL	37°C, 24h	Methanol extract shows potency against all the bacteria with activity range from 54 % to 81% total activity.	[3]
				B. subtilis	62.5/250 µg/mL	37°C, 24h		
				E. coli	125/500 µg/mL	37°C, 24h		
				E. chrysanthemi	125/250 µg/mL	37°C, 24h		
				X. phaseoli	250/250 µg/mL	37°C, 24h		
Cryptomeria japonica	all parts	Methanol	Phenolic compounds	*Staphylococcus aureus*	1600 µg/mL	37°C, 24h	*C. japonica* methanol extracts each part except for pollen showed strong activities	[4]
A. japonica	leaves	Methanol	Phenolic compounds	*B. subtilis*	250/500 µg/mL	37°C, 24h	Methanol extract shows potency against all the bacteria with activity range from 54 to 81% total activity.	[3]
				E. coli	250/500 µg/mL	37°C, 24h		
				X. phaseoli	250/500 µg/mL	37°C, 24h		
C. sempervirens	aerial part	Methanol	Phenolic compounds	*Enterococcus feacalis*	250/250 µg/mL	37°C, 24 h	Plant extract shows good antibacterial activity	[5]
				Staphlococcus aureus	125 /125 µg/mL	37°C, 24 h		
				K. pneumonia	62.5/62.5 µg/mL	37°C, 24 h		
				Pseudomonas aeruginosa	125/125 µg/mL	37°C, 24 h		
				Salmonella indica	125/125 µg/mL	37°C, 24 h		
Juniperus communis	berry	Alcohol / Water	Flavanoids (quercetin, rutin, apigenin) chlorogenic acid	*Penicillium hirsutum, A. niger*	50 µL/mL	37°C, 84 h.	Berry extract shows good antifungal activity	[6]
Juniperus communis	leaves	hexane	Phenolic compounds	*B. subtilis*	40 µL/mL	37°C, 24 h	Hexane extract shows maximum zone of inhibition against bacteria with 16-21 mm	[7]
				E. coli	40 µL/mL	37°C, 24 h		
				A. tumefaciens	40 µL/mL	37°C, 24 h		
				E. chrysanthemi	40 µL/mL	37°C, 24 h		
				X. phaseoli	250/500 µg/mL	37°C, 84 h		

Picea abies	bark	diethyl ether	Hydroxystilbene, resveratrol, isorhapontigenin, piceatannol	*Antrodia sinuosa, Phlebiopsis gigantea, Serpula himantioides, Antrodia xantha, G. sepiarium, Fomitopsis pinicola, Coniophora puteana, Heterobasidion parviporum* and *Serpula lacrymans*	8 and 15 mg/L	NF	Compound piceatannol and isorhapontigenin as compared to resveratrol showed best antifungal activity in brown rot fungi	[8]
		Ethanol / Water	Stilbenoids	*S. aureus* and *Candida albicans*	33.33 mg/mL	35°C, 24 h; 25°C, 48–72 h.	*Picea abies* bark extracts exhibited growth inhibition against *S. aureus* and *C. albicans*	[9]
P. mariana	bark	Water	Phenolic compounds	*E. coli*	1.67 mg/mL	37°C, 24 h.	OPF displayed best antimicrobial Activity as compared to WE	[10]
		Oligomeric proanthocyanidins fraction (OPF)		*E. coli*	0.83 / 4.44 mg/mL	37°C, 24 h.		
P. smithiana	bark	Methanol/ethanol	Alkaloids, flavonoids, tannins and phenols	*A. tumefaciens, B. subtilis, E. coli, E. chrysanthemi* and *X. phaseoli*	31.25-250 μg/mL	37°C, overnight	Both the extracts of *P. smithiana* showed a significant zone of inhibition ranging from 9-19 mm (ZOI). The results showed that methanol extract has more antimicrobial potential than ethanol, highest in *Agrobacterium tumefaciens*. The lowest value of MIC and MBC were recorded against *A. tumefaciens* in methanol extract 31.25 μg/mL and 62.5 μg/mL respectively.	[11]
P. gerardiana	bark	Ethanol	flavonoids	*P. aeruginosa*	1500 μg/mL	37°C, 24 h	PG extract shows antibacterial zone of inhibition with 10.5 and	[12]

							antifungal activity with 15.01mm zone of inhibition	
P. roxburghii	bark	Ethanol	flavonoids	Bacteria	(µg/mL)	37°C, 24 h	PR extract shows antibacterial and antifungal activity with 10.2 to 13.2 and 15.3 mm zone of inhibition respectively	[12]
				P. aeruginosa	NF			
				S. aureus	1000-1500 µg/mL			
				E. coli	NF			
				K. pneumonia	1000-1500 µg/mL			
				C. albicans	1500 µg/mL			
P. wallichiana	bark	Ethanol	flavonoids	Bacteria		37°C, 24 h	PW extract shows antibacterial activity with zone of inhibition 10.12 to 14.21 mm and antifungal with 18.93 mm	[12]
				P. aeruginosa	1000-1500 µg/mL			
				S. aureus	1000-1500 µg/mL			
				K. pneumonia	1000-1500 µg/mL			
				C. albicans	1500 µg/mL	25°C, 72h		
Pinus wallichiana	leaves	Methanol	flavonoids, phenols	*A. tumefaciens*	250/500 µg/mL	37°C, 24h	Extract shows significant antibacterial activity	[3]
				B. subtilis	500/500 µg/mL	37°C, 24h		
				E. coli	500/na µg/mL	37°C, 24h		
				E. chrysanthemi	500/500 µg/mL	37°C, 24h		
				X. phaseoli	500/na µg/mL	37°C, 24h		
Taxus baccata	leaves	Ethanol	flavonoids, phenols	*A. tumefaciens*	250/500 µg/mL	37°C, 24h	Extract shows significant antibacterial activity	[3]
				B. subtilis	250/500 µg/mL	37°C, 24h		
				E. coli	250/500 µg/mL	37°C, 24h		
				E. chrysanthemi	250/500 µg/mL	37°C, 24h		
				X. phaseoli	250/500 µg/mL	37°C, 24h		
Thuja occidentalis	leaves	Methanol	flavonoids, phenols	*P. auregenosa, Salmonella, B. subtilis, Bacillus cereus, A. niger* and *Candida krusie*	100, 150, 200 and 250 mg/mL	37°C, 24-48 h	Shows good antimicrobial activity	[13]
Taxus wallichiana	Leaf, stem	Methanol	Polyphenols, flavanoids, terpenoids	*S. aureus, E. coli*	LC_{50} (µg/mL) Leaves =601.17 Stem= (3.56×10^{8})	37°C, overnight	Stem of *T. wallichiana* showed significant zone of inhibition against gram positive bacteria while the leaf of *T. wallichiana*	[14]

	did not show significant zone of inhibition against both gram positive and gram negative bacteria

NF-Not found; na–not active

Reference

1. Freitas, A.M.; Almeida, M.T.R.; Andrighetti-Fröhner, C.R.; Cardozo, F.T.G.S.; Barardi, C.R.M.; Farias, M.R.; Simões, C.M.O. Antiviral activity-guided fractionation from Araucaria angustifolia leaves extract. *J. Ethnopharmacol.* **2009**, *126*, 512–517, doi:10.1016/j.jep.2009.09.005.
2. Banerjee, S.; Das, A.; Chakraborty, P.; Suthindhiran, K.; Jayasri, M.A. Antioxidant and antimicrobial activity of Araucaria cookii and Brassaia actinophyla. *Pakistan J. Biol. Sci.* **2014**, *17*, 715–719, doi:10.3923/pjbs.2014.715.719.
3. Joshi, S.; Sati, S.C.; Kumar, P. Antibacterial potential and ethnomedical relevance of Kumaun Himalayan Gymnosperms. *J. Phytopharm.* **2016**, *5*, 190–200.
4. Horiba, H.; Nakagawa, T.; Zhu, Q.; Ashour, A.; Watanabe, A.; Shimizu, K. Biological activities of extracts from different parts of cryptomeria japonica. *Nat. Prod. Commun.* **2016**, *11*, 1337–1342, doi:10.1177/1934578x1601100939.
5. Selim, S.A.; Adam, M.E.; Hassan, S.M.; Albalawi, A.R. Chemical composition, antimicrobial and antibiofilm activity of the essential oil and methanol extract of the Mediterranean cypress (Cupressus sempervirens L.). *BMC Complement. Altern. Med.* **2014**, *14*, 1–8, doi:10.1186/1472-6882-14-179.
6. Fierascu, I.; Ungureanu, C.; Avramescu, S.M.; Cimpeanu, C.; Georgescu, M.I.; Fierascu, R.C.; Ortan, A.; Sutan, A.N.; Anuta, V.; Zanfirescu, A.; et al. Genoprotective, antioxidant, antifungal and anti-inflammatory evaluation of hydroalcoholic extract of wild-growing Juniperus communis L. (Cupressaceae) native to Romanian southern sub-Carpathian hills. *BMC Complement. Altern. Med.* **2018**, *18*, 1–15, doi:10.1186/s12906-017-2066-8.
7. Sati, S.C.; Joshi, S.; Campus, D.S.B. Antibacterial potential of leaf extracts of Juniperus communis L. from Kumaun Himalaya. *African J. Microbiol. Res.* **2010**, *4*, 1291–1294.
8. Hedenström, E.; Fagerlund Edfeldt, A.; Edman, M.; Jonsson, B.G. Resveratrol, piceatannol, and isorhapontigenin from Norway spruce (Picea abies) debarking wastewater as inhibitors on the growth of nine species of wood-decaying fungi. *Wood Sci. Technol.* **2016**, *50*, 617–629, doi:10.1007/s00226-016-0814-4.
9. Välimaa, A.L.; Raitanen, J.E.; Tienaho, J.; Sarjala, T.; Nakayama, E.; Korpinen, R.; Mäkinen, S.; Eklund, P.; Willför, S.; Jyske, T. Enhancement of Norway spruce bark side-streams: Modification of bioactive and protective properties of stilbenoid-rich extracts by UVA-irradiation. *Ind. Crops Prod.* **2020**, *145*, 112150, doi:10.1016/j.indcrop.2020.112150.
10. St-Pierre, A.; Blondeau, D.; Bourdeau, N.; Bley, J.; Desgagné-Penix, I. Chemical Composition of Black Spruce (Picea mariana) Bark Extracts and Their Potential as Natural Disinfectant. *Ind. Biotechnol.* **2019**, *15*, 219–231, doi:10.1089/ind.2019.0007.
11. Kumar, P. Ph ton. **2015**.
12. Sharma, A.; Goyal, R.; Sharma, L. Potential biological efficacy of Pinus plant species against oxidative, inflammatory and microbial disorders. *BMC Complement. Altern. Med.* **2016**, *16*, 1–11, doi:10.1186/s12906-016-1011-6.
13. Deepika Tekaday; Reena Antony; Sourabh Jain Antimicrobial, antioxidant and phytochemical investigation of Thuja occidentalis (Arbor vitae) leave extract. *GSC Biol. Pharm. Sci.* **2020**, *12*, 108–116, doi:10.30574/gscbps.2020.12.3.0292.
14. Subba, B. Analysis of Phytochemical Constituents and Biological Activity of Taxus Wallichiana Zucc. Dolakha District of Nepal. *Int. J. Appl. Sci. Biotechnol.* **2018**, *6*, 110–114, doi:10.3126/ijasbt.v6i2.20410.
